# Integrating 3D imaging, GWAS, and single-cell transcriptome approaches to elucidate root system architecture in *Populus*

**DOI:** 10.1093/plphys/kiaf432

**Published:** 2025-09-26

**Authors:** Jingjing Li, Wenhao Bo, Chenhao Bu, Jiaxuan Zhou, Peng Li, Menglei Wang, Yuepeng Song, Qing Liu, Yousry A El-Kassaby, Deqiang Zhang

**Affiliations:** State Key Laboratory of Tree Genetics and Breeding, National Engineering Research Center of Tree Breeding and Ecological Restoration, Key Laboratory of Genetics and Breeding in Forest Trees and Ornamental Plants, Ministry of Education, College of Biological Sciences and Biotechnology, Beijing Forestry University, Beijing 100083, China; State Key Laboratory of Tree Genetics and Breeding, National Engineering Research Center of Tree Breeding and Ecological Restoration, Key Laboratory of Genetics and Breeding in Forest Trees and Ornamental Plants, Ministry of Education, College of Biological Sciences and Biotechnology, Beijing Forestry University, Beijing 100083, China; State Key Laboratory of Tree Genetics and Breeding, National Engineering Research Center of Tree Breeding and Ecological Restoration, Key Laboratory of Genetics and Breeding in Forest Trees and Ornamental Plants, Ministry of Education, College of Biological Sciences and Biotechnology, Beijing Forestry University, Beijing 100083, China; State Key Laboratory of Tree Genetics and Breeding, National Engineering Research Center of Tree Breeding and Ecological Restoration, Key Laboratory of Genetics and Breeding in Forest Trees and Ornamental Plants, Ministry of Education, College of Biological Sciences and Biotechnology, Beijing Forestry University, Beijing 100083, China; State Key Laboratory of Tree Genetics and Breeding, National Engineering Research Center of Tree Breeding and Ecological Restoration, Key Laboratory of Genetics and Breeding in Forest Trees and Ornamental Plants, Ministry of Education, College of Biological Sciences and Biotechnology, Beijing Forestry University, Beijing 100083, China; State Key Laboratory of Tree Genetics and Breeding, National Engineering Research Center of Tree Breeding and Ecological Restoration, Key Laboratory of Genetics and Breeding in Forest Trees and Ornamental Plants, Ministry of Education, College of Biological Sciences and Biotechnology, Beijing Forestry University, Beijing 100083, China; State Key Laboratory of Tree Genetics and Breeding, National Engineering Research Center of Tree Breeding and Ecological Restoration, Key Laboratory of Genetics and Breeding in Forest Trees and Ornamental Plants, Ministry of Education, College of Biological Sciences and Biotechnology, Beijing Forestry University, Beijing 100083, China; CSIRO Agriculture and Food, Black Mountain, Canberra, Australian Capital Territory, Canberra ACT 2601, Australia; Department of Forest and Conservation Sciences, Faculty of Forestry, Forest Sciences Centre, University of British Columbia, Vancouver, British Columbia BC V6T 1Z4, Canada; State Key Laboratory of Tree Genetics and Breeding, National Engineering Research Center of Tree Breeding and Ecological Restoration, Key Laboratory of Genetics and Breeding in Forest Trees and Ornamental Plants, Ministry of Education, College of Biological Sciences and Biotechnology, Beijing Forestry University, Beijing 100083, China; College of Horticulture and Landscape Architecture, Beijing Vocational College of Agriculture, Beijing 102442, China

## Abstract

Roots are essential for nutrient uptake and structural stability in trees. Despite their critical role, the genetic determinants underlying root system architecture (RSA) remain poorly understood. In this study, we employed an integrated approach combining automated 3-dimensional (3D) spatial imaging, multiomics analyses, genetic transformation, and molecular experiments to investigate the genetic architecture and regulatory networks governing RSA in Simon poplar (*Populus simonii*). Here, using a panel of 303 *P. simonii* accessions collected from different geographical regions in China, we performed a genome-wide association study (GWAS) on 96 RSA traits and identified S-phase kinase-associated protein 2B (PsiSKP2B) as a candidate gene colocalized by 6 traits. By integrating the findings from GWAS, transcriptome, and single-cell RNA-seq (scRNA-seq) analyses, we identified *PsiSKP2B* as a key regulator of meristematic tissue cells involved in lateral root (LR) development. Overexpression of *PsiSKP2B* in 84k (*Populus alba* × *Populus glandulosa*) had a substantial effect on RSA traits, increasing the number and density of LRs by 65.9% and 98.6%, respectively, compared with wild-type plants. Our in vitro and in vivo assays revealed that PsiSKP2B modulates LR development by interacting with WUSCHEL-RELATED HOMEOBOX 4 (PsiWOX4) or ZINC FINGER HOMEODOMAIN 9 (PsiZHD9), both of which are specifically expressed in atrichoblast cells, thereby activating a regulatory feedback loop. These findings highlight an atrichoblast-dependent regulatory mechanism through which PsiSKP2B governs LR development. Our study not only introduces an advanced image recognition methodology for quantifying RSA traits in *P. simonii* but also provides a comprehensive multiomics framework for elucidating the genetic and molecular basis of RSA.

## Teaser

Integrating advanced imaging and multiomics clarifies regulatory mechanisms of poplar root system architecture, offering scientific support to enhance root traits and advance sustainable forestry.

## Introduction

In land plants, the root system functions as the primary organ for responding to soil conditions and facilitating the uptake of water, mineral elements, and other essential nutrients (Lynch 1995). The morphological characteristics of the root system are crucial determinants of a plant's adaptive capacity (Abramoff and Finzi 2015). Root system architecture (RSA) encompasses a diverse array of spatial and morphological features such as root angles, specific root area, root diameter, root length density, total root length, root branching, and root biomass (Ranjan et al. 2022; Tian et al. 2023). Recent technological advancements have led to the development of high-throughput platforms and semiautomated tools for temporal assessment of RSA traits (Narisetti et al. 2019). When characterizing root system phenotypes, it is essential to employ metrics that are highly resistant to errors to ensure the reliability of the acquired data. Currently, several holistic indicators are employed to evaluate RSA. For example, the primary roots of Chinese white poplar (*Populus tomentosa*) and black locust (*Robinia pseudoacacia*) primarily fulfill structural and anchorage functions, while secondary roots are preferentially developed for resource exploration and spatial expansion (Di et al. 2018; Feng et al. 2024). Despite these advancements, existing studies often provide merely qualitative descriptions of complex morphological traits, and detailed examinations of microscopic morphologies remain substantially limited.

The advent of the phenomics era, driven by advances in imaging and computational technologies, has revolutionized the ability to record plant morphological phenotypes (Singh et al. 2018; Roitsch et al. 2019). These high-throughput, precise, rapid, and nondestructive techniques primarily employ photographic methods for data acquisition, making a substantial departure from traditional quantitative metrics to image-based analyses (Fraas and Luthen 2015). Current methodologies for investigating RSA include field observations, greenhouse studies, nuclear magnetic resonance (NMR), and micro-root canals (Galkovskyi et al. 2012; Mairhofer et al. 2015; Sousa-Neto et al. 2018). For example, phenotypic traits such as mean root diameter and lateral root (LR) branching angle have been successfully quantified in rice (*Oryza sativa*) grown in agar-based transparent media (Galkovskyi et al. 2012). Despite the proliferation of experimental systems and software primarily designed for analyzing crop roots (Seethepalli et al. 2021; Shukla et al. 2023), substantial challenges remain in adapting these technologies for tree species. For instance, tree species develop their root systems predominantly from adventitious roots through cuttings, which subsequently form a taproot system composed of multiple adventitious roots. Current software often struggle to accurately identify the initial root node in such configurations. Consequently, the measurement and analysis of root traits in tree species remain relatively underdeveloped.

LR development is a critical process of plant root formation and environmental adaptation, tightly regulated by multiple genetic and signaling pathways (Gao et al. 2023; Wu et al. 2024). In recent years, the ubiquitin–proteasome system (UPS) in plant development has garnered increasing attention. The Skp1–Cullin–F-box (SCF) complex, a core component of the E3 ubiquitin ligase, plays a pivotal role in a variety of biological processes by mediating the ubiquitination and degradation of target proteins (Callis and Vierstra 2000; Hellmann and Estelle 2002). Its function in LR formation has been particularly well characterized, with extensive studies demonstrating its regulatory importance in this developmental process (Xu et al. 2002; Chapman and Estelle 2009; Yu et al. 2015). SKP2B, a homolog of the human cell cycle S-phase kinase-associated protein2 (Skp2), is part of the SCF complex that recruits phosphorylated *ARABIDOPSIS THALIANA* HOMOLOG OF E2F C (AtE2Fc) to regulate cell division (Ren et al. 2008; Manzano et al. 2012). *AtSKP2* binds to S-PHASE KINASE-ASSOCIATED PROTEIN 1 (Skp1) via its F-box domain and recognizes specific substrate proteins through other structural domains (leucine-rich repeat [LRRs] proteins, WD40-DOMAIN 1 [WD40]). In *Arabidopsis* (*Arabidopsis thaliana*), *SKP2B*, which is expressed throughout all stages of LR development (Manzano et al. 2014), negatively modulates the cell cycle and LR formation by repressing meristematic and founder cell divisions (Manzano et al. 2012). Although research on *SKP2B* has been extensively conducted in *A. thaliana*, its role in RSA development in woody plants remains largely unexplored, indicating a substantial area for further investigation to elucidate the regulatory mechanisms underlying RSA.

Transcriptomic analyses have provided a wealth of information for identifying key regulators of LR development (De Smet et al. 2008; Ramakrishna et al. 2019). Detailed temporal staging and analyses of the various steps involved in LR formation have not only identified additional regulators but have also highlighted the complexity of the regulatory pathways (Voß et al. 2015). Further complicating this analysis is the fact that LR development is noncell-autonomous, involving multiple cell types that each play distinct roles and activate a variety of genetic networks throughout the process (Vermeer et al. 2014; Porco et al. 2016). Cell sorting analyses, aimed at dissecting tissue-specific signals during LR development, have been limited due to technical challenges (Chen et al. 2023). The exceptionally small size of root regions undergoing fate transitions makes such analyses highly complex and impractical. Single-cell RNA-sequencing (scRNA-seq) offers a powerful alternative for profiling transcriptomes at the resolution of individual cells, substantially reducing the amount of tissue required compared with cell sorting (Jean-Baptiste et al. 2019). To date, single-cell analyses of root tissue have predominantly focused on several key areas: gene expression profiles in the primary root, comparative transcriptome changes between hair and nonhair cells, processes of endodermal differentiation, and the regenerative response of the primary root meristem following injury (Shulse et al. 2019; Denyer et al. 2019; Shahan et al. 2022).

Simon poplar (*Populus simonii*), a prominent native tree species in northern China, is renowned for its cold and drought resistance, rapid growth rate, and robust root system (Bi et al. 2022). In finer-textured soils, *P. simonii* exhibits enhanced LR proliferation, whereas in coarser soils, the LRs display increased diameter and length (Zhang et al. 2016). These roots often extend vertically or obliquely downward, enhancing the plant's access to deeper water reserves (Tariq et al. 2024). Therefore, elucidating the regulatory mechanism of LR development and mining key functional genes are important for coping with environmental stress and optimizing plant growth. In this study, we investigated the genetic underpinnings of RSA by performing GWAS on 96 root traits of 303 unrelated accessions, within a natural population of *P. simonii*. By integrating GWAS, transcriptome, and scRNA-seq, we identified that PsiSKP2B, a part of the SCF complex, positively regulates LR development by recognizing and binding to PsiWOX4 and PsiZHD9, mediating their ubiquitination and proteasomal degradation. Single-cell transcriptomic analysis revealed that *PsiSKP2B* is predominantly expressed in meristematic cells, whereas *PsiWOX4* and *PsiZHD9* are mainly expressed in atrichoblast cells. Additionally, the enrichment of auxin (indole-3-acetic acid [IAA])-related genes in meristematic and atrichoblast cells implied that auxin signaling may play a substantial role in *PsiSKP2B*-mediated development. In summary, PsiSKP2B plays a crucial role in LR development by modulating the protein stability of WUSCHEL-RELATED HOMEOBOX 4 (PsiWOX4) and ZINC FINGER HOMEODOMAIN 9 (PsiZHD9), a process likely regulated by the auxin signaling pathway. This study not only elucidates the molecular mechanism of *PsiSKP2B* in LR development but also provides an important theoretical basis for *Populus* root improvement and stress tolerance breeding.

## Results

### High correlation of 96 traits of root system microphenotype

**Figure 1.**
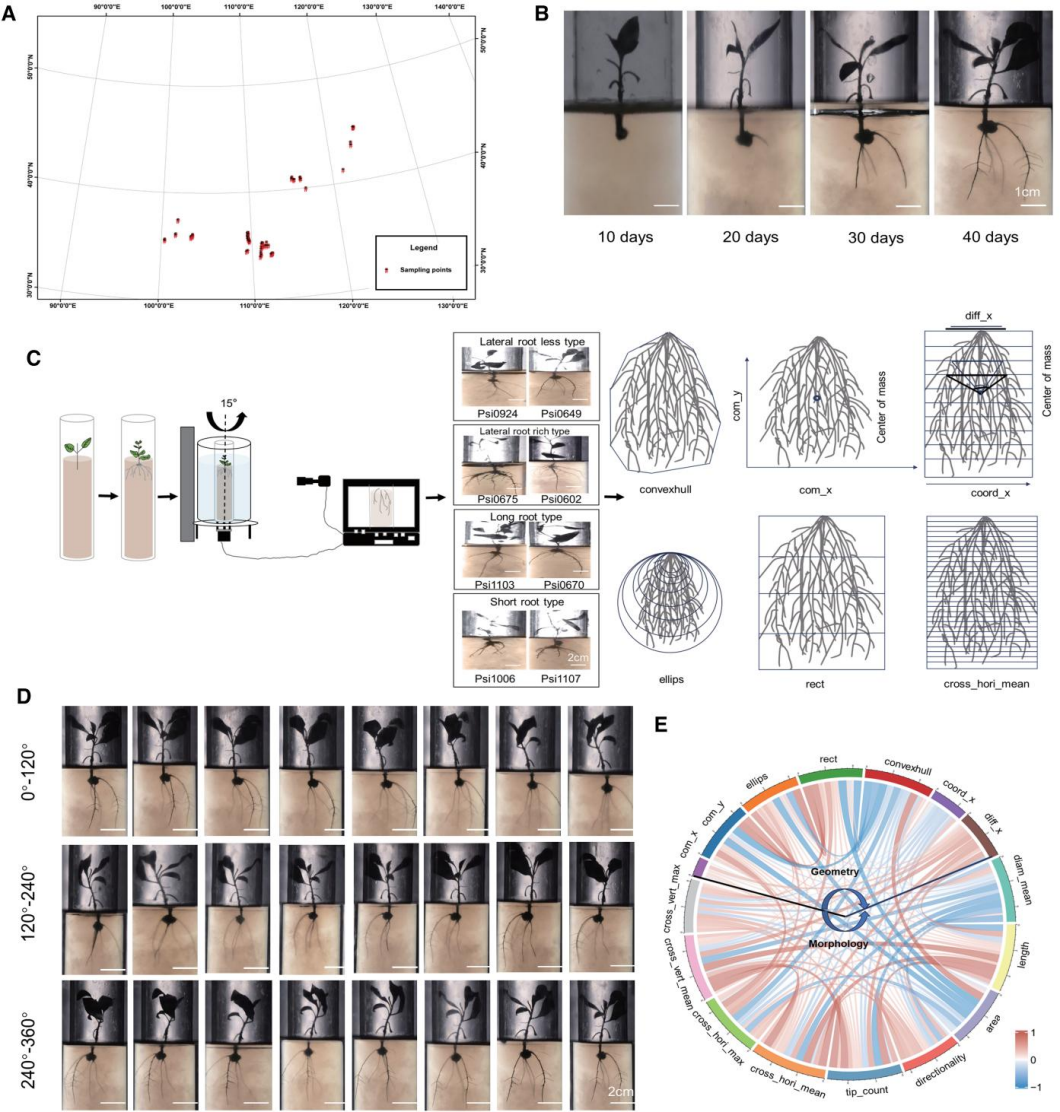
Comprehensive workflow for root phenotypic data acquisition. **A)** Geographic distributions of 303 *P. simonii* individuals. Red triangles represent sample collection points. Different colors represent altitude. **B)** Changes of *Populus* RSA over intervals of 10, 20, 30, and 40 d. Scale bar = 1 cm. **C)** Simple process for photographing root system from multiple angles. The apparatus is set to automatically capture images at 15°, generating 24 two-dimensional images per accession. Representative raw images (accession: Psi0924, Psi0649, Psi0675, Psi0602, Psi1103, Psi0670, Psi1006, Psi1107) show root architectural variation across rotation angles (scale bar =2 cm). Diagram of image processing workflow: raw images, background subtraction, root segmentation, 3D reconstruction. Representative root phenotypes (convexhull, ellips, rect, com_x, com_y, coord_x, cross_hori_mean, diff_x) are shown. Convexhull: the minimal convex area encapsulating the root system; Ellips: the proportion of root area encompassed by ellipses of varying sizes, centered at the apex of the root system; Rect: the proportion of root area distributed across 4 stratified layers of the root system; Com_x: relative coordinate of the center of mass of the root system (*x*); Com_y: relative coordinate of the center of mass of the root system (*y*); Coord_x: *x*-coordinates of the shape of the root system in 10 equal layers; Cross_hori_mean: mean root detected when scanning the root system with a horizontal line at the depth; Diff_x: difference between *x* values for each layer of the shape. Describe the angle of the root system in each layer. **D)** Multiangle photographs of *Populus* roots (40 d) are shown for demonstrating the morphology and structure of the root system. Scale bar = 2 cm. Multiangle images of 303 accessions with 3 biological replicates were acquired, totaling 24 images per accession. **E)** Phenotypic correlation of 16 macrophenotypes in *P. simonii* natural population. The thickness and color of the ribbons correlate to the correlation of macrophenotypes. Negative value indicates negative correlations, and positive value indicates positive correlations. Com_x, com_y, ellips, rect, convexhull, coord_x, and diff_x are geometry descriptors. Diam_mean, length, area, directionality, tip_count, cross_hori_mean, cross_hori_max, cross_vert_mean, and cross_vert_max are morphology descriptors.

To investigate the phenotypic diversity of root development within the natural population of Simon poplar (*P. simonii*), we quantified 96 phenotypic traits across 303 distinct accessions (Fig. 1A). Notably, extensive phenotypic variations were observed until 40 d within the population, when its spatial distribution became distinctly visible (Fig. 1B). Multiangle images of 303 accessions with 3 biological replicates were acquired, totaling 24 images per accession (Fig. 1, C and D). With RiaRoot software, we ultimately generated a comprehensive dataset comprising 96 root microphenotypes and 16 macrophenotypes. These included 7 root structural features, such as com_x (relative coordinates of the center of mass of the root system), com_y (relative coordinate of the center of mass of the root system [*y*]), ellips (percentage root area in varying ellipsoidal sizes centered on the root apex), rect (quartile distribution of root area), convexhull (area of the smallest convex shape encapsulating the root system), coord_x (*x*-coordinate characterizing the root system's morphology), and diff_x (differential in *x*-values across each root layer, the larger the *x*, the greater the angle of entrapment). Additionally, 9 root morphological features were analyzed to capture organ-scale traits linked to water uptake and mechanical stability, including diam_mean (mean diameter of the root object in the image), length (length of the skeleton of the root system), area (projected area of the root system), directionality (mean direction of the root segments in the root system), tip_count (number of end branches in the root system skeleton), cross_hori_mean (mean roots detected when scanning the root system with a horizontal line at the depth), cross_hori_max (max roots identified via a horizontal scanning at depth), cross_vert_mean (mean number of roots detected when scanning the root system with a vertical line), and cross_vert_max (max number of roots detected when scanning the root system with a vertical line). The selected traits collectively reflect adaptive strategies in root systems. LR density (cross_hori_mean) and root surface area (area) enhance soil exploration for phosphate and nitrate (Postma et al. 2014). For instance, higher cross_hori_mean in deep soil layers correlates with improved nitrogen acquisition in low-nitrogen environments (Lynch et al. 2023). Root system convex hull area (convexhull) and center of mass (com_y) influence plant anchorage; shallower com_y values are associated with lodging resistance in windy environments (Shah et al. 2019). Traits like diff_x (angle dispersion) and directionality reflect phenotypic plasticity in response to soil heterogeneity. These traits comprehensively characterize the structural and morphological features of the root system in multiple dimensions (Supplementary Table S1).

Correlation analyses showed a high degree of correlation among several phenotypic traits (Supplementary Fig. S1). For example, the correlation coefficient between coord_x8 and coord_x9 was as high as 0.99 (Supplementary Fig. S1C), indicating that these traits may be governed by the identical or closely related genetic regulatory mechanisms. In addition, the 5 traits rect, diff_x, ellips, cross_hori_mean, and cross_hori_max showed predominantly positive correlations with each other (Supplementary Fig. S1, A and B, D to F), suggesting that they may have a synergistic effect during root development. For instance, a higher diff_x value, which reflects a greater root angle, may enhance root expansion in the soil, thereby increasing the number of LR (cross_hori_mean) and the maximum root count (cross_hori_max). Among the 16 macrophenotypes, the strongest correlation observed was 0.99 (Fig. 1E), while the correlation between rect and ellips was 0.98 (Fig. 1E). These highly correlated traits may be associated with colocalization of key genes, implying that they may be regulated by the same or closely linked genes.

### GWAS reveals key genes regulating RSA in *Populus*

We analyzed a germplasm diversity panel comprising 303 accessions of *P. simonii*. Genomes of these accessions were sequenced using Illumina technology, and raw sequence data were obtained. A total of 639,988 single-nucleotide polymorphisms (SNPs) with a missing rate of no more than 1 and a minor allele frequency (MAF) of ≥0.05 were identified and used for downstream analyses. We conducted a comprehensive analysis of the population structure in our genome-wide association study (GWAS) dataset to account for potential confounding effects. Using the 639,988 high-quality SNPs, we performed principal component analysis (PCA) to identify the genetic structure of the 303 accessions of *P. simonii* (Supplementary Fig. S2). The PCA results revealed that the first principal component (PC1) explained 65.1% of the variance and the second principal component (PC2) explained 7.4% of the variance (Supplementary Fig. S2B). Kinship matrix analysis further confirmed low genetic relatedness (95.78% of kinship coefficients < 0.05; Supplementary Fig. S3), supporting the panel's suitability for GWASs without substantial stratification bias. The genetic structure analysis, PCA, and phylogenetic studies clearly show the genetic distances between accessions, highlighting the presence of 3 major monophyletic clades within the population (Supplementary Fig. S2).

**Figure 2.**
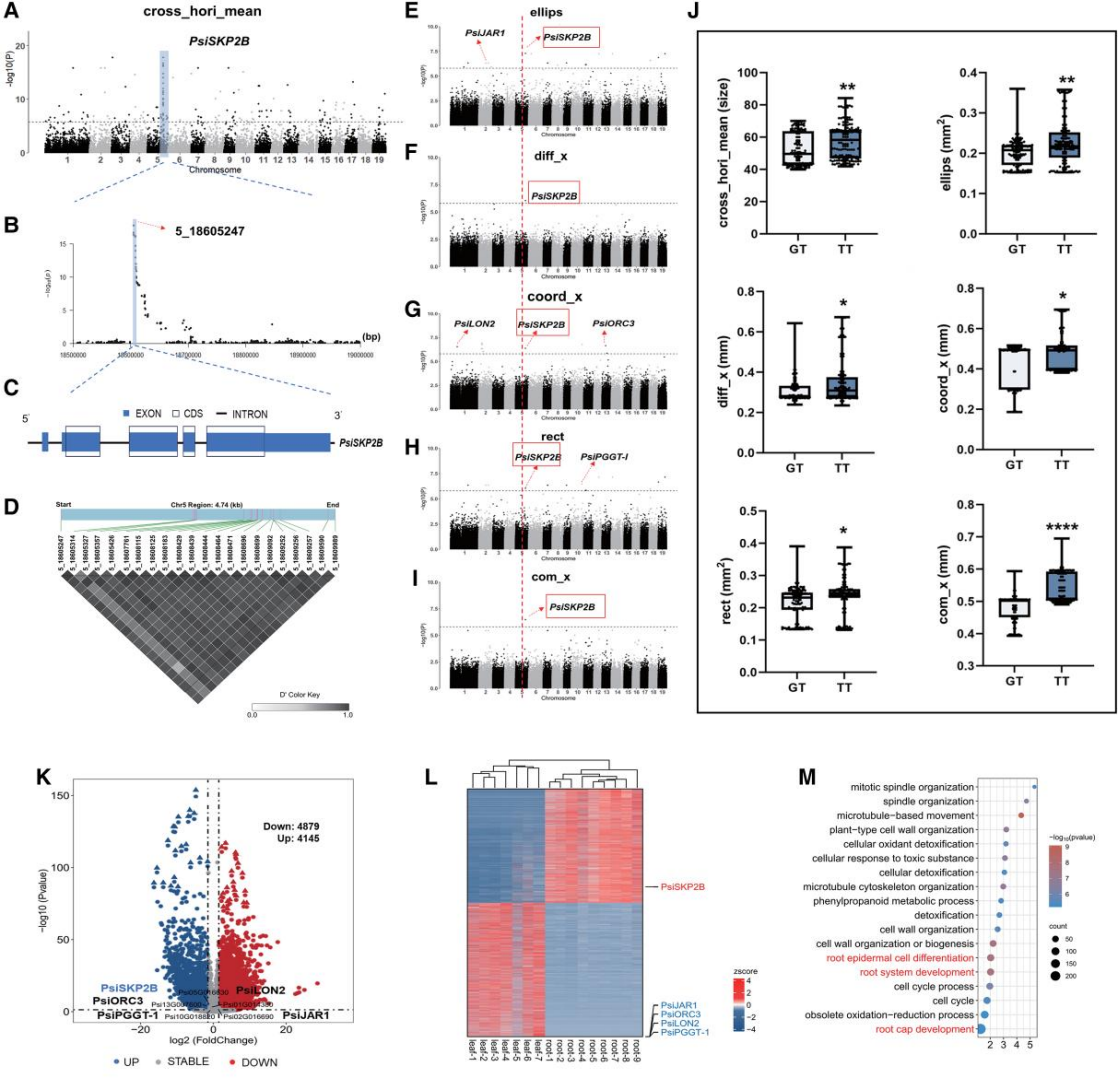
*PsiSKP2B* is a potential selective gene that contributes to RSA of *P. simonii*. **A)** Manhattan plots depict GWAS results of *PsiSKP2B* for the “cross_hori_mean” trait. A black dotted line marks Bonferroni-adjusted significance threshold (*P* = 1.56 × 10^−6^). The *x*-axis represents genomic coordinates across all chromosomes, and the *y*-axis shows −log₁₀(*P*-values) from MLM-based association tests (639,988 SNPs). **B)** Local Manhattan plot of *PsiSKP2B* genomic region on chromosome 5 (18.5 to 19 mb). SNPs in candidate genes, as identified by GWAS, are shown as a red arrow. The *x*-axis shows genomic coordinates, and the *y*-axis displays −log₁₀(*P*-values) from GWAS. **C)** Schematic diagram of the gene structure of *PsiSKP2B*. Blue boxes represent exons, blank boxes represent CDSs (total length = 1,134 bp), and lines represent introns. **D)** LD values among all SNPs in *PsiSKP2B*. Heatmap displays pairwise LD values (*D*′) among 22 SNPs identified within the 4.74-kb *PsiSKP2B* locus (Chr5: 18,605,247–18,609,989). Color gradient from white (*D*′ = 0) to gray (*D*′ = 1) indicates the strength of allelic association between SNPs. **E** to **I)** Manhattan plot of GWAS for 5 traits, which are ellips **E)**, diff_x **F)**, crood_x **G)**, rect **H)**, and com_x **I)**. A black dotted line marks Bonferroni-adjusted significance threshold (*P* = 1.56 × 10^−6^). The red dashed lines denote the *PsiSKP2B* locus corresponding to ellips **E)**, diff_x **F)**, crood_x **G)**, rect **H)**, and com_x **I)**. The *x*-axis represents genomic coordinates across all chromosomes, and the *y*-axis shows −log₁₀(*P*-values) from MLM-based association tests (639,988 SNPs). **J)** SNP contribution to phenotypic variations. The data are shown as boxplots: The center line represents the median; the box limits indicate the upper (75th) and lower (25th) quartiles; the whiskers extend to 1.5 times the interquartile range from the quartiles; and points show outliers. Boxplots show 6 traits (cross_hori_mean, ellips, diff_x, coord_x, rect, and com_x) in 2 genotypes. Data are shown as mean ± SEM with jittered individual data points. Different asterisk (*) numbers indicate statistically significant differences (1-way ANOVA followed by post hoc Tukey test; * for *P* < 0.05, ** for *P* < 0.01, and *** for *P* < 0.001); ns, not significant. **K)** Volcano plots show differential expression of genes (DEG) in LR and leaf of *P. simonii* (*P* < 0.05, |log2 fold change| > 1). Blue dots represent upregulated genes. Red dots represent downregulated genes, and gray dots represent genes with stable expression levels. Genes (*PsiSKP2B*) in the blue dots represent significantly upregulated genes. Genes (*PsiLON2*, *PsiORC3*, *PsiPGGT-1*, and *PsiJAR1*) in the gray dots show no significant differential expression. Triangles indicate the top 20 DEGs based on fold-change criteria. **L)** Tissue-specific expression heatmap. Heatmap shows specific expression of candidate genes expressed in LR and leaf tissues. The LR samples were meticulously gathered from distinct regions of the main root, specifically the upper (root-1, root-2, and root-3 were collected from the basal 1/3 of the main root), middle (root-4, root-5, and root-6 were collected from the central 1/3 of the main root), and lower sections (root-7, root-8, and root-9 were collected from the apical 1/3 of the main root, including the root tip and meristematic zone). Numbers indicate biological replicates within each positional zone. Leaf samples (leaf-1, leaf-2, leaf-3, leaf-4, leaf-5, leaf-6, and leaf-7) were used as controls. The color scale in the heatmap represents gene expression levels, which are normalized using *Z*-score standardization during data processing. Red tones indicate higher-than-average expression (positive *Z*-scores). Blue tones indicate lower-than-average expression (negative *Z*-scores). White colors correspond to expression near the mean (*Z*-score ≈ 0). **M)** GO functional enrichment analysis of brown module. The top 20 GO term categories are shown. The *y*-axis shows biological processes, and the *x*-axis indicates gene ratios. Bubble sizes represent gene numbers. In GO enrichment analyses, color is frequently employed to signify the level of significance. Lower *P*-values indicate greater significance, whereas higher *P*-values suggest lesser significance.

Based on the 639,988 high-confidence SNPs, a GWAS was conducted on the 96 traits employing a mixed linear model (MLM). A total of 150 SNP loci were significantly associated with the 96 traits (*P* ≤ 1.56 × 10^−6^ [1/*n*], Bonferroni test), among which SNP Chr5_18605247 was associated with 6 traits. Candidate genes were identified within 2 kb upstream and downstream of the associated SNP loci, based on the linkage disequilibrium (LD) decay in the whole genome (Supplementary Fig. S2D). A total of 72 expressed genes were detected among the 96 traits (Supplementary Table S2). Some of the previously reported root development genes were also included in the GWAS results, including S-phase kinase-associated protein 2B (PsiSKP2B), JASMONATE RESISTANT 1 (PsiJAR1), LON PROTEASE 2 (PsiLON2), ORIGIN RECOGNITION COMPLEX SUBUNIT 3 (PsiORC3), and protein geranylgeranyltransferase type-I (PsiPGGT-I) (Supplementary Fig. S4A) (Lingard and Bartel 2009; Manzano et al. 2012; Chen et al. 2013; Cai et al. 2014). Notably, SNP Chr5_18605247, located 40 bp upstream of *PsiSKP2B*, showed significant associations with 6 phenotypic traits (com_x, coord_x, cross_hori_mean, diff_x, ellips, and rect), with the strongest association observed for cross_hori_mean (Fig. 2, A to I; *P* ≤ 1.56 × 10^−6^ [1/*n*], Bonferroni test). These findings suggest that *PsiSKP2B* may play an important role in regulating LR development. The frequency of the T allele, which is associated with the 6 phenotypic traits, increased, while the frequency of heterozygous alleles decreased, further indicating a strong genetic association between this allele and the observed phenotypic traits (Fig. 2J). It is widely acknowledged that *PsiSKP2B* is a putative ortholog of *AtSKP2* (*AT1G77000*) in *Arabidopsis* (*A. thaliana*) (Supplementary Fig. S4B) and functions as an F-box ubiquitin ligase, thereby fostering cell division and founder cell division (Ren et al. 2008; Manzano et al. 2012). Additionally, the genes *PsiJAR1* (SNP Chr2_12910083), *PsiLON2* (SNP Chr1_12444305), *PsiORC3* (SNP Chr13_6933516), and *PsiPGGT-I* (SNP Chr10_19520430) exhibited significant associations with ellips, coord_x, and rect, respectively (Fig. 2, E, G, and H; *P* ≤ 1.56 × 10^−6^ [1/*n*], Bonferroni test). The protein encoded by *PsiJAR1* is a jasmonic acid (JA)-amido synthetase, belonging to the GH3 protein family (Delfin et al. 2022). It interacts with light, glucose, and auxin signaling pathways to finely regulate the branching angle of *Arabidopsis* LRs (Sharma et al. 2022). *PsiLON2* codes for a Lon protease-like protein, showing severe defects in IBA-induced LR formation observed in LON2 mutants (Lingard and Bartel 2009). *PsiPGGT-I* is predicted to encode the beta subunit of geranylgeranyl transferase (GGT-IB), which is involved in abscisic acid (ABA)-mediated and auxin signaling pathways (Johnson et al. 2005). *PsiORC3* is transcriptionally regulated during the cell cycle, expressed in regions of active cell proliferation, including the primary root tip, stem base, LR primordium, emerged LR primordium, LR tip, young shoot, anther, and ovary. Notably, knockdown of *OsORC3* plants lacked LRs (Chen et al. 2013). While these candidate genes are essential for cell division, LR formation, meristem development, and hormone signaling, they exhibit weaker co-association with multiple traits and lack evidence of direct genetic association with multiple phenotypic traits compared with *PsiSKP2B* (del Pozo et al. 2002; Manzano et al. 2012; Supplementary Table S3).

### The specific expression of *PsiSKP2B* in the root

To comprehensively dissect the process of root development and validate the functions of candidate genes identified through GWAS, we performed comprehensive transcriptomic sequencing of LR and leaf tissues (control) in *P. simonii*. A total of 76 million reads were aligned to the *P. simonii* genome, yielding an average alignment rate of 71.2% (Supplementary Table S4). Differential expression analysis identified 9,024 differentially expressed genes (DEGs), including 4,879 downregulated and 4,145 upregulated genes (Fig. 2K; Supplementary Table S5). To explore the biological processes associated with LR development, we performed gene ontology (GO) enrichment analyses on the DEGs. Substantial enrichment was observed in the molecular function category for terms related to LR morphogenesis (GO:0010102), LR development (GO:0048527), and postembryonic root development (GO:0010101) (Supplementary Table S6). By integrating GWAS and transcriptomic data, we found that *PsiSKP2B* was specifically expressed in root tissues and significantly upregulated (*P*-value < 0.05, adjusted for the false discovery rate; Fig. 2, K and L). In contrast, *PsiORC3*, *PsiPGGT-I*, *PsiJAR1*, and *PsiLON2* were expressed in the root but were not identified as DEGs, suggesting that these genes may play fundamental or nonspecific roles in root development (Fig. 2, K and L).

To identify key factors associated with LR development, we employed weighted gene co-expression network analysis (WGCNA) to analyze 9,024 DEGs (after deleting extremely low expression or low variable coefficient values) between LR and leaf tissues. In WGCNA, modules represent clusters of highly interconnected genes, where genes within the same cluster exhibit strong correlation coefficients. A total of 12 co-expression modules were identified, each containing between 46 and 2,322 DEGs (Supplementary Table S7). The interrelationships between 12 modules were visualized in a cluster dendrogram (Supplementary Fig. S5, A and B). Notably, the brown module, comprising 1,703 genes including *PsiSKP2B*, was substantially enriched for genes involved in root cap development (GO:0048829), root system development (GO:0022622), and root epidermal cell differentiation (GO:0010053) (Fig. 2M). The involvement of *PsiSKP2B* in this module provides further evidence of its close association with RSA.

To validate the reliability of the transcriptome results, we conducted reverse-transcription quantitative PCR (RT-qPCR) on *PsiSKP2B*, *PsiORC3*, *PsiPGGT-I*, *PsiJAR1*, and *PsiLON2* (Supplementary Fig. S5C). The RT-qPCR results demonstrated that only the DEG *PsiSKP2B* exhibited the same expression trends as observed in the RNA-seq analysis, confirming the accuracy of the RNA-seq results. To better understand the evolutionary relationships and functional conservation of SKP2 proteins, we performed a molecular phylogenetic analysis. We collected the sequences of known SKP2 proteins from *Arabidopsis*, including SKP2A (AtSKP2;1, AT1G21410), SKP2B (AtSKP2;2, AT1G77000), and several other SKP2 putative paralogs (AT5G22660, AT5G44490, AT2G42720). Sequence similarity analysis revealed that PsiSKP2B shares 71.6% similarity with SKP2A and 72.2% similarity with SKP2B (Supplementary Fig. S6A). Additionally, phylogenetic analysis placed PsiSKP2B in the same clade as AT1G77000 across diverse plant species (*Arabidopsis*, Robusta [*Coffea canephora*], pepper [*Capsicum annuum*], potato [*Solanum tuberosum*], rice [*O. sativa*]), confirming their putatively orthologous relationship (Supplementary Fig. S6B).

### Clusters comprise the major cell types in the root

**Figure 3.**
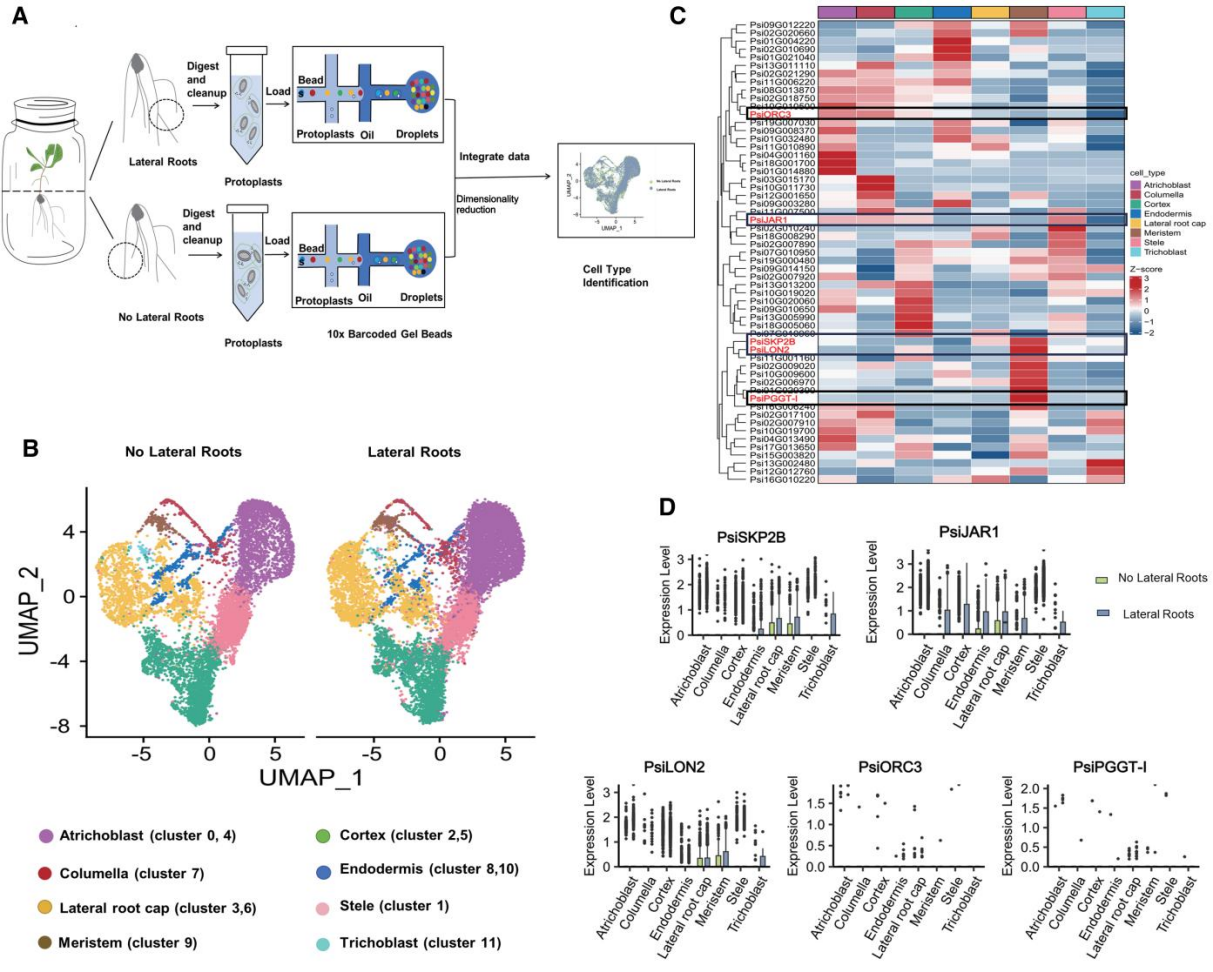
Cluster annotation of *Populus* root tips and *PsiSKP2B* is expressed in meristem. **A)** Workflow of the single-cell RNA-sequencing (scRNA-seq) in *Populus* root tips. Protoplasts isolated from the LR sample and no LR sample are loaded separately into a 10× Genomics Chromium Controller for single-cell transcriptomic analysis. **B)** UMAP visualization of 8 cell types in *Populus* root tip. Each dot denotes a single cell. Colors denote corresponding cell type. Each cell type is derived from 1 or 2 clusters. **C)** Expression levels of candidate genes in 8 cell types, identified through GWAS. Expression is color coded to indicate positive and negative expression levels. **D)** Expression patterns of GWAS-identified candidate genes in cell types of the LR sample and no LR sample. The data are shown as boxplots: the center line represents the median; the box limits indicate the upper (75th) and lower (25th) quartiles; the whiskers extend to 1.5 times the interquartile range from the quartiles; and points show outliers. Columns in green represent the no LR sample, and blue columns represent the LR sample. Identified cell types are enumerated on the horizontal axis.

To elucidate the cell-type-specific expression patterns of *PsiSKP2B* during LR development and to gain cellular-level insights into its regulatory mechanisms, we performed scRNA-seq analyses. Protoplasts were isolated from LR at 2 developmental stages (no LRs and LRs). Following prefiltering of the sequencing data, a total of 10,000 cells were retained for analysis (Fig. 3A). The results showed that 76.6% of the sequencing reads were successfully mapped to the *P. simonii* genome and 97.1% of the barcodes were validated (Supplementary Table S8). We captured 9,090 cells for the no LR sample, with a median of 1,508 genes detected per cell. For the LR sample, we captured 12,414 cells, with a median of 1,319 genes per cell (Supplementary Table S8).

Within the resolution spectrum of 0.2 to 1.6, a resolution setting of 0.4 was determined to be optimal for generating a balanced number of cell clusters. It is adept at discerning substantial biological distinctions, such as variations in cell types or states, and mitigates the risk of spuriously dividing cells of the same type due to technical variability (Supplementary Fig. S7). Unsupervised analyses using the Uniform Manifold Approximation and Projection (UMAP) or t-SNE algorithms with a resolution of 0.4 successfully grouped cells into 12 distinct clusters (Becht et al. 2019; Zhou and Jin 2020) (Supplementary Fig. S8, A and B). To assess the conservation across the 2 root developmental stages, scRNA-seq data were integrated and analyzed jointly. The integrative clustering revealed that the 12 cell-type clusters aligned well and matched between the 2 root states (Supplementary Fig. S8C). The distribution of cell numbers across 12 clusters ranged from 78 to 4,230, reflecting the heterogeneous cellular composition of the root (Supplementary Table S8). To identify additional marker genes, the top 5 DEGs from each of the 12 cell clusters were used to generate a heatmap, illustrating their diverse expression profiles (*P* < 0.05, |log2FC| ≥ 0.58) (Supplementary Table S9; Supplementary Fig. S8, D and E).

The cell types within the 12 clusters were assessed by an analysis of homologs of known cell-type marker genes according to protein sequence similarity, confirmed in other scRNA-seq studies, or the model plant *A. thaliana* (Denyer et al. 2019; Gala et al. 2021) (Supplementary Table S10). The comparative analysis enabled the identification of 8 biological cell types and functions within the *Populus* root tip: atrichoblast (cluster 0 and cluster 4), stele (cluster 1), cortex (cluster 2 and cluster 5), LR cap (cluster 3 and cluster 6), columella (cluster 7), endodermis (cluster 8 and cluster 10), meristem (cluster 9), and trichoblast (cluster 11) (Supplementary Tables S10 and S11; Fig. 3B). These findings demonstrate that the scRNA-seq approach successfully captured all major root tissue types (Fig. 3B). Among the identified cell types, atrichoblast and cortex were notably abundant, comprising 32.2% and 22.1% of the total cell count, respectively (Supplementary Fig. S8F; Supplementary Table S12). Furthermore, gene expression analysis revealed substantial overlap in expressed genes between the no LR sample and LR sample within larger cell-type clusters, including atrichoblast (78.1%), endodermis (67.6%), stele (61.5%), and meristem (55.4%) (Supplementary Table S13). In contrast, the trichoblast exhibited the lowest percentage of root-state-specific genes (23.8%), which is in accordance with the marked morphology divergence observed in this cell type between different root conditions (Hendriks et al. 2022).

### Characterization of GWAS-identified candidate genes in each cell type

In this study, 57 of the 72 candidate genes identified by GWAS showed substantial expression in different cell types of the root system (Fig. 3C). We further investigated the expression patterns of genes implicated in LR development, including *PsiSKP2B*, *PsiORC3*, *PsiPGGT-I*, *PsiJAR1*, and *PsiLON2* (Fig. 3D). These genes exhibited distinct tissue-specific expression profiles, emphasizing their nuanced roles in root development. For example, *PsiJAR1* was abundantly expressed in various cell types, including columella, cortex, endodermis, LR cap, meristem, and trichoblast (Fig. 3D). Notably, it was most prominently expressed in the cortex of the LR sample region, suggesting a substantial role in this region's response to LR development (Fig. 3D). In addition, *PsiLON2* and *PsiSKP2B* were primarily expressed in the meristem, with *PsiLON2* being highly expressed in trichoblasts in the LR sample region and hardly expressed in the no-LR-sample region (Fig. 3D). Consistent with *PsiJAR1*, both *PsiLON2* and *PsiSKP2B* displayed elevated expression levels in the LR sample (Fig. 3D), indicating their potential involvement in maintaining root architecture and functionality under stable growth conditions. In contrast, *PsiORC3* and *PsiPGGT-I* exhibited extremely low expression levels throughout the root system, suggesting that their roles in root development might be limited or possibly dependent on specific environmental or developmental contexts that warrant further investigation (Fig. 3D). Additionally, *PsiSKP2B* expression in the LR cap was second only to its expression in meristematic cells, indicating the potential involvement in LR development.

To elucidate the physiological distinctions among the various cell types in the *P. simonii* root system, we analyzed the expression patterns of cell-type-specific marker genes (Supplementary Fig. S8E). GO analyses facilitated the identification of DEGs that were upregulated in each cell type (|log2FC| ≥ 0.58 and *P*-value < 0.05, relative to the other 7 cell types), and functions of marker genes in each cell type were investigated (Supplementary Fig. S9; Supplementary Table S11). For example, genes in the LR cap were predominantly associated with xenobiotic stimulus (GO:0009410), multidimensional cell growth (GO:0009825), and fungal attack (GO:0050832) (Supplementary Fig. S9D). In meristematic cell clusters, there was a substantial enrichment of genes involved in responding to organonitrogen compounds (GO:0010243) and auxin (GO:0009733) (Supplementary Fig. S9F). Atrichoblast cells exhibited a high level of genes associated with the biosynthetic process of organonitrogen compound (GO:1901566) and auxin signaling pathway (GO:0009737) (Supplementary Fig. S9C). The genes expressed in endodermis cells were related to ribosome biogenesis (GO:0042254) and the ATP metabolic process (GO:0046034) (Supplementary Fig. S9E). Concurrently, the genes ubiquitously expressed across the 8 identified cell types were predominantly involved in responses to abiotic stimuli (GO:0009628) and cellular biosynthetic processes (GO:0044249) (Supplementary Table S11).

### *PsiSKP2B* positively regulates LR development in *Populus*

To investigate the role of *PsiSKP2B* in *Populus* LR development, we generated RNA interference (RNAi) and overexpressing (OE) transgenic plants. The OE transgenic plants were generated using the pBI121 vector that contained the *PsiSKP2B* coding sequence (CDS) under the control of the 35S promoter. Among the 11 OE lines generated, 3 lines (OE-1, OE-9, and OE-11) exhibiting the highest *PsiSKP2B* expression levels were selected for further analysis (Supplementary Fig. S10A). For the RNAi lines, transgenic poplar plants were constructed using the pEGOEP35S-H vector that contained 2 inverted copies of the cDNA fragment of *PsiSKP2B* driven by the 35S promoter. From the 6 RNAi lines obtained, 3 lines (Ri-1, Ri-2, and Ri-6) with the lowest *PsiSKP2B* expression levels were chosen for subsequent studies (Supplementary Fig. S10B).

**Figure 4.**
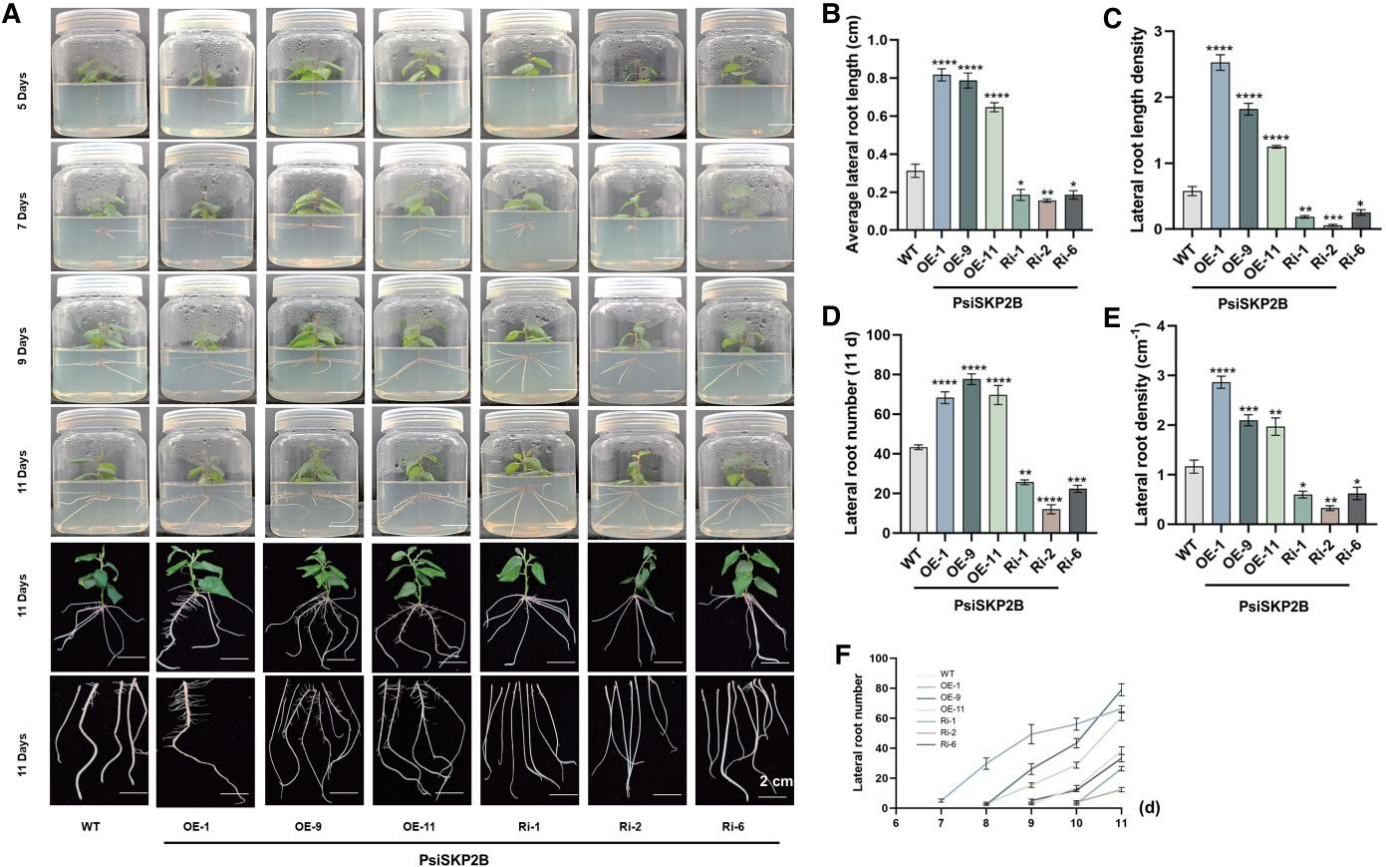
*PsiSKP2B* positively regulates LR development under normal conditions. **A)** Root system morphology of *PsiSKP2B* transgenic and WT plants. Root system morphology changes differ between *PsiSKP2B*-OE and *PsiSKP2B*-RNAi (Ri) plants and WT plants cultured on MS medium at 25 °C, with a 12-h light/dark cycle and a light intensity of 2,000 l× for 11 d. Scale bar = 2 cm. **B** to **F)** Six phenotypic measurements of *PsiSKP2B* transgenic and WT plants. Average LR length **B)**, LR length density **C)**, LR number (11 d) **D)**, LR density **E)**, LR number **F)** of *PsiSKP2B* transgenic and WT plants. Data are shown as mean ± SEM with 3 biological replicates. Different asterisk (*) numbers indicate statistically significant differences (1-way ANOVA followed by post hoc Tukey test; * for *P* < 0.05, ** for *P* < 0.01, *** for *P* < 0.001, and **** for *P* < 0.0001); ns, not significant.

To assess the involvement of *PsiSKP2B* in LR development, 6 independently generated transgenic lines (OE-1, OE-9, OE-11, Ri-1, Ri-2, and Ri-6) were selected and observed LR phenotypes (Fig. 4A). *PsiSKP2B*-OE lines showed significantly enhanced LR growth, with 2.2-fold higher LR length density and 1.3-fold longer average LR length (*P* < 0.0001, Tukey test; Fig. 4, B and C). Eleven-day-old RNAi plants showed 53.8% fewer LRs than WT (Fig. 4D). In contrast, overexpression of *PsiSKP2B* significantly increased the LR number (65.9%) and density of LRs (98.6%) and promoted the growth of LR (*P* < 0.0001, Tukey test; Fig. 4, D and E). LR formation was observed 1 to 2 d earlier in OE-1, OE-9, and OE-11 lines in an 11-d course tracking (Fig. 4F). To investigate the effect of *PsiSKP2B* on auxin levels, we performed auxin quantification in *PsiSKP2B*-OE and *PsiSKP2B*-RNAi transgenic lines. The results showed that auxin levels were significantly higher (16.6%) in *PsiSKP2B*-OE lines compared with WT plants, while *PsiSKP2B*-RNAi lines exhibited significantly lower (7.1%) auxin levels (*P* < 0.0001, Tukey test; Supplementary Fig. S11). We also found that the LR traits of *PsiSKP2B*-OE lines matched the original root trait (cross_hori_mean) in GWAS (Fig. 2A). Taken together, these results suggest that *PsiSKP2B* is involved in regulating LR development positively, potentially through modulating auxin levels.

### Combining DEG and WGCNA to identify the interacting proteins of PsiSKP2B

**Figure 5.**
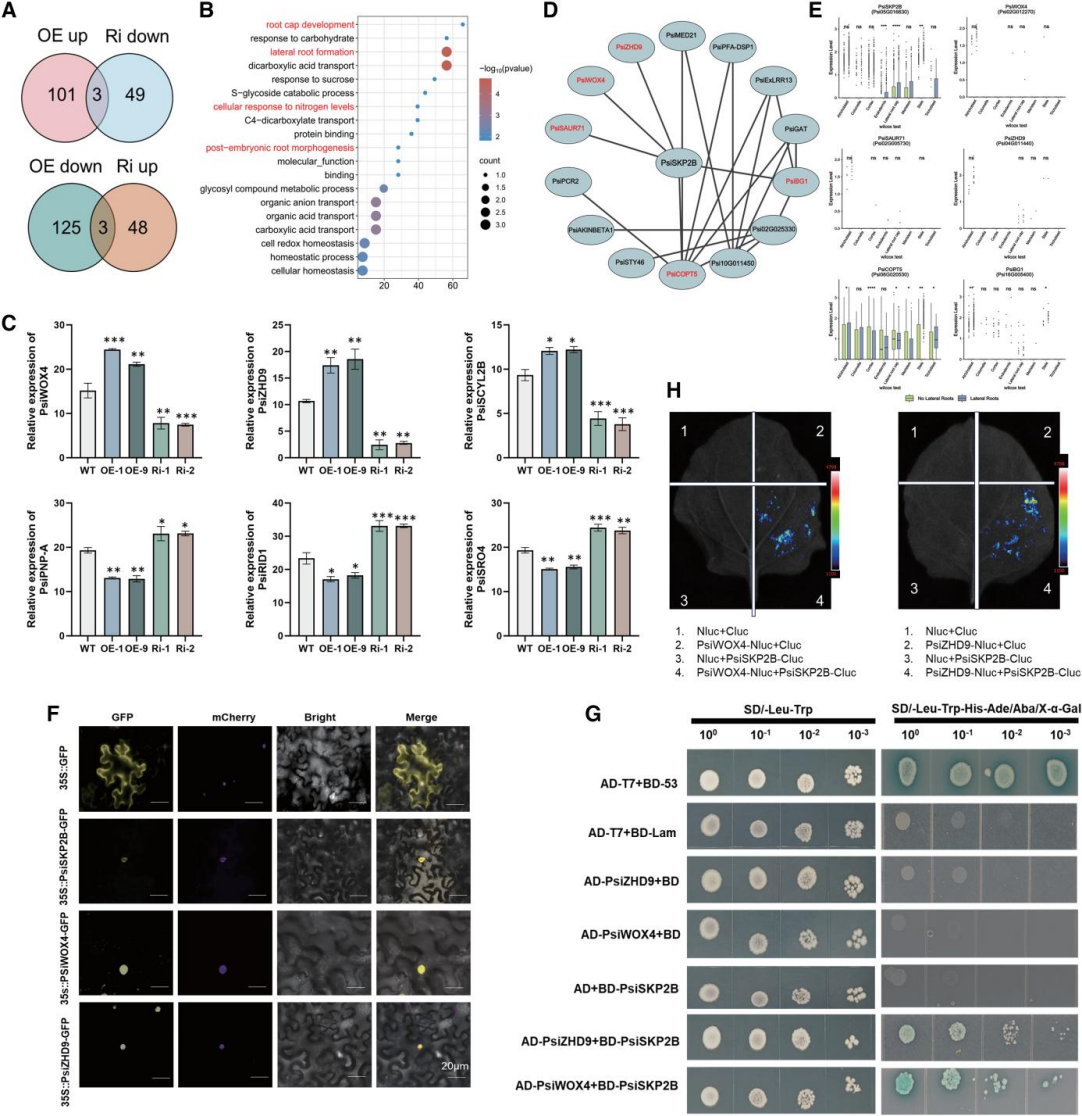
Predicting and validating *PsiSKP2B* target genes. **A)** Venn diagrams represent DEGs regulated by *PsiSKP2B*. **B)** GO enrichment of the significantly upregulated genes in *PsiSKP2B*-OE lines (*P*-value < 0.05, adjusted for the false discovery rate). The top 20 GO term categories are shown. The *y*-axis shows biological processes, and the *x*-axis indicates gene ratios. Bubble sizes represent gene numbers. **C)** Expression levels of *PsiWOX4*, *PsiZHD9*, *PsiSCYL2B*, *PsiPNP-A*, *PsiRID1*, and *PsiSRO4* in WT and transgenic poplar lines, as determined by RT-qPCR. Data are shown as mean ± SEM with 3 biological replicates. Different asterisk (*) numbers indicate statistically significant differences (1-way ANOVA followed by post hoc Tukey test; * for *P* < 0.05, ** for *P* < 0.01, and *** for *P* < 0.001); ns, not significant. **D)** Gene co-expression network of the turquoise module. The node and edge size are proportional to the core. **E)** Boxplot graph shows expression levels of the genes associated with *PsiSKP2B* in the turquoise module in the LR sample and no LR sample. The data are shown as boxplots: the center line represents the median; the box limits indicate the upper (75th) and lower (25th) quartiles; the whiskers extend to 1.5 times the interquartile range from the quartiles; and points show outliers. Identified cell types are enumerated along the horizontal axis. Asterisks mark significant differences using unpaired 2-sided Wilcoxon test: ns, *P* > 0.05; *, *P* < 0.05; **, *P* < 0.01; ***, *P* < 0.001; ****, *P* < 0.0001. **F)** Subcellular localization of *PsiSKP2B*, *PsiWOX4*, and *PsiZHD9* in transiently expressed *N. benthamiana* leaves. Scale bar = 20 μm. **G)** PsiSKP2B interacts with PsiWOX4 and PsiZHD9 in yeast, respectively. The pGBKT7-lam (BD-lam) is used as a negative control, while pGBKT7-53 (BD-53) is used as a positive control. The positive transformants are cultured on SD-Trp-His-Ade-Leu/X-α-gal/Aba medium. The negative transformants are cultured on SD-Trp-Leu medium. **H)** A split-luciferase complementation assay shows the interaction of *PsiSKP2B-*Cluc with *PsiWOX4-*Nluc or *PsiZHD9*-Nluc in *N. benthamiana* leaves. Nluc with *PsiSKP2B*-Cluc, Cluc with *PsiWOX4*-Nluc, Cluc with *PsiZHD9*-Nluc, and Cluc with Nluc are used as negative controls.

To investigate the transcriptional regulatory network of *PsiSKP2B* in response to regulating LR, we performed RNA-seq analysis on poplar roots from *PsiSKP2B*-OE and *PsiSKP2B*-RNAi lines grown under normal conditions. Based on the marked expression differences observed in these lines (OE-1, OE-9, Ri-1, and Ri-2) during RT-qPCR analysis, the 4 transgenic lines were subsequently selected for further differential expression analysis (Supplementary Fig. S10). Comparative analysis between *PsiSKP2B*-OE and WT plants identified 232 DEGs, including 104 significantly upregulated and 128 significantly downregulated genes (*P*-value < 0.05, adjusted for the false discovery rate) (Fig. 5A; Supplementary Fig. S12A; Supplementary Table S14). *PsiSKP2B*-RNAi lines exhibited 103 DEGs, with 51 significantly upregulated and 52 significantly downregulated genes (*P*-value < 0.05, adjusted for the false discovery rate) (Fig. 5A; Supplementary Fig. S12B; Supplementary Table S15). These DEGs were primarily associated with biological processes such as LR formation (GO:0010311), glycosyl compound metabolic process (GO:1901657), carbohydrate metabolic process (GO:0005975), and developmental processes (GO:0032502) (Fig. 5B; Supplementary Table S16; Supplementary Fig. S12, C to E). These findings suggested that *PsiSKP2B* plays a pivotal role in modulating transcriptional networks involved in LR development and related metabolic pathways.

To identify core genes regulated by *PsiSKP2B*, we screened for DEGs that could respond to both *PsiSKP2B*-OE and *PsiSKP2B*-RNAi (Fig. 5A; Supplementary Fig. S12, A and B). The result of Venn analysis showed that 6 DEGs were co-regulated by *PsiSKP2B*-OE and *PsiSKP2B*-RNAi (Fig. 5A). Among these, 3 DEGs (*PsiWOX4*, *PsiZHD9*, SCY1-like protein 2 B [*PsiSCYL2B*]) were positively regulated by *PsiSKP2B*, as their transcription was significantly enhanced in *PsiSKP2B*-OE lines but markedly reduced in *PsiSKP2B*-RNAi lines compared with the control (*P*-value < 0.05, adjusted for the false discovery rate) (Fig. 5C). In contrast, the expression of 3 DEGs (SIMILAR TO RCD ONE 4 [*PsiSRO4*], PLANT NATRIURETIC PEPTIDE A [*PsiPNP-A*], ROOT INITIATION DEFECTIVE 1 [*PsiRID1*]) was negatively regulated by *PsiSKP2B*, and its silencing promoted their expression (Fig. 5C). The transcript levels of the aforementioned DEGs were tightly regulated by *PsiSKP2B*, with opposite effects on gene transcription observed upon overexpression or silencing of *PsiSKP2B*. Given the established positive regulatory role of *PsiSKP2B* in LR development (Fig. 4, B to F), we focused on 3 DEGs (*PsiWOX4*, *PsiZHD9*, and *PsiSCYL2B*) that were positively regulated by *PsiSKP2B*, as evidenced by their upregulation in *PsiSKP2B*-OE lines and downregulation in *PsiSKP2B*-RNAi lines. These genes may represent direct targets of *PsiSKP2B* in *Populus* roots, providing insights into the molecular mechanisms underlying *PsiSKP2B*-mediated LR development.

To reveal key factors associated with LR development, WGCNA was used to analyze 232 DEGs (after deleting extremely low expression and low variable coefficient values) between *PsiSKP2B*-OE and WT. Among these, 126 correlated genes were grouped into a turquoise module that specifically associated with *PsiSKP2B*. By focusing on genes with the highest connectivity (top 20 weight values), we constructed a co-expression network centered on *PsiSKP2B* (Supplementary Tables S17 and S18; Fig. 5D). This network highlighted 5 pivotal genes (*PsiZHD9*, SMALL AUXIN UPREGULATED 71 [*PsiSAUR71*], *PsiWOX4*, COPPER TRANSPORTER 5 [*PsiCOPT5*], and beta-1,3-glucanase 1 [*PsiBG1*]) implicated in various biological functions, such as auxin response, copper transport, and WUSCHEL-related homeobox regulation (Tan and Irish 2006; Garcia-Molina et al. 2011; Qiu et al. 2020; Wang et al. 2020). They were predominantly expressed in the atrichoblast (Fig. 5E; Supplementary Fig. S13). In addition, we discovered that *PsiSKP2B*-OE markedly enhanced the transcription of these DEGs (*PsiWOX4*, *PsiZHD9*, *PsiSCYL2B*) in contrast to the control, while *PsiSKP2B*-RNAi significantly diminished their expression (*P* < 0.001, Tukey test; Fig. 5C). Integrating the results from DEG analysis and WGCNA, we propose that PsiWOX4 and PsiZHD9 proteins may physically interact with PsiSKP2B, respectively. These results indicate that PsiWOX4 and PsiZHD9 are positively regulated by PsiSKP2B during the development of *Populus* roots and are most likely direct targets of PsiSKP2B.

### PsiSKP2B interacts directly with PsiWOX4 or PsiZHD9

To investigate the mechanism underlying *PsiSKP2B*-mediated LR development, we attempted to identify whether PsiSKP2B physically interacts with PsiWOX4 or PsiZHD9. Subcellular localization experiments indicated that *PsiWOX4* and *PsiZHD9* were localized to the nucleus, consistent with the localization pattern of *PsiSKP2B* (Fig. 5F). Yeast 2-hybrid (Y2H) assays showed that the co-transformed yeast of *PsiSKP2B* with *PsiWOX4* and *PsiZHD9*, respectively, could grow normally on the yeast-deficient solid medium (-Trp/-His/-Ade/-Leu) containing 400 ng/mL competitive inhibitor Aureobasidin A (AbA) (Fig. 5G). Thus, we speculated that *PsiWOX4* and *PsiZHD9* were potential interactors for *PsiSKP2B*-mediated LR development.

To confirm this hypothesis, we evaluated the physical interaction between PsiSKP2B and PsiWOX4 or PsiZHD9 using a luciferase complementation imaging assay. After the vector pairs (*PsiWOX4*-Nluc and *PsiSKP2B*-Cluc, *PsiZHD9*-Nluc and *PsiSKP2B*-Cluc) were co-expressed in *Nicotiana benthamiana* leaves, we detected luciferase activity in the combination, but not in the negative control combinations (Fig. 5H). The interactions of PsiSKP2B with PsiZHD9 and PsiWOX4 were further confirmed by a coimmunoprecipitation (co-IP) assay (Supplementary Fig. S14A). Taken together, these in vivo and in vitro results suggest that PsiSKP2B physically interacts with PsiWOX4 and PsiZHD9.

To determine whether PsiWOX4 and PsiZHD9 were ubiquitination substrates of PsiSKP2B, we transiently expressed *PsiZHD9*-Flag and *PsiWOX4*-Flag or co-expressed them with *PsiSKP2B*-GFP in *N. benthamiana* leaves and detected the in vivo ubiquitination of PsiZHD9 and PsiWOX4 proteins. In the presence of *PsiSKP2B*-GFP, we observed that PsiZHD9 and PsiWOX4 were ubiquitinated by PsiSKP2B (Supplementary Fig. S14B). Furthermore, the addition of the proteasome inhibitor MG132 further attenuated the rate of *PsiZHD9*-Flag or *PsiWOX4*-Flag degradation in protein extracts. However, no notable degradation of control GFP proteins was observed using the same plant extracts (Supplementary Fig. S14, B and C). These results confirmed that PsiSKP2B is specifically involved in degrading PsiZHD9 and PsiWOX4 proteins.

In addition, *PsiSKP2B* was expressed in meristematic cells, and *PsiWOX4* and *PsiZHD9* were mainly expressed in atrichoplast cells (Fig. 5E; Supplementary Fig. S13). To validate the expression patterns of these candidate genes in vivo, we performed in situ hybridization and were able to identify marker genes for poplar root cell types. For example, *Psi03G016300* was specifically expressed in meristematic cells, and *Psi14G013520* was specifically expressed in atrichoplast cells, which is consistent with our annotation results (Supplementary Fig. S15, A and B). These findings further support the specific expression of *PsiSKP2B* in meristematic cells, as well as the expression patterns of *PsiWOX4* and *PsiZHD9* in atrichoplast cells (Supplementary Fig. S15, A and B). Additionally, we visualized UMAP plots for all 8 major cell types using their respective marker genes (Supplementary Fig. S15C), providing a comprehensive overview of the cellular taxonomy. The integration of in situ hybridization with marker gene visualization substantially enhances the reliability of cell-type annotation.

*PsiSKP2B* is known to be expressed in meristematic cells, *PsiZHD9* and *PsiWOX4* are expressed in atrichoblasts, and both are enriched for auxin (Supplementary Fig. S15, A and B; Supplementary Fig. S9, C and F). This spatial arrangement provides a basis for *PsiSKP2B* to modulate *PsiZHD9*/*PsiWOX4* via the auxin signaling pathway, thereby influencing LR development. For *PsiSKP2B*, it is possible that auxin signaling activates the ubiquitin ligase activity of PsiSKP2B or that auxin modulates the molecular mechanisms underlying the interaction between PsiSKP2B and PsiZHD9/PsiWOX4, thereby enabling PsiSKP2B to more efficiently ubiquitinate and degrade these proteins.

## Discussion

### PsiSKP2B governs LR development by interacting with PsiWOX4 and PsiZHD9 from meristem to atrichoblast

**Figure 6.**
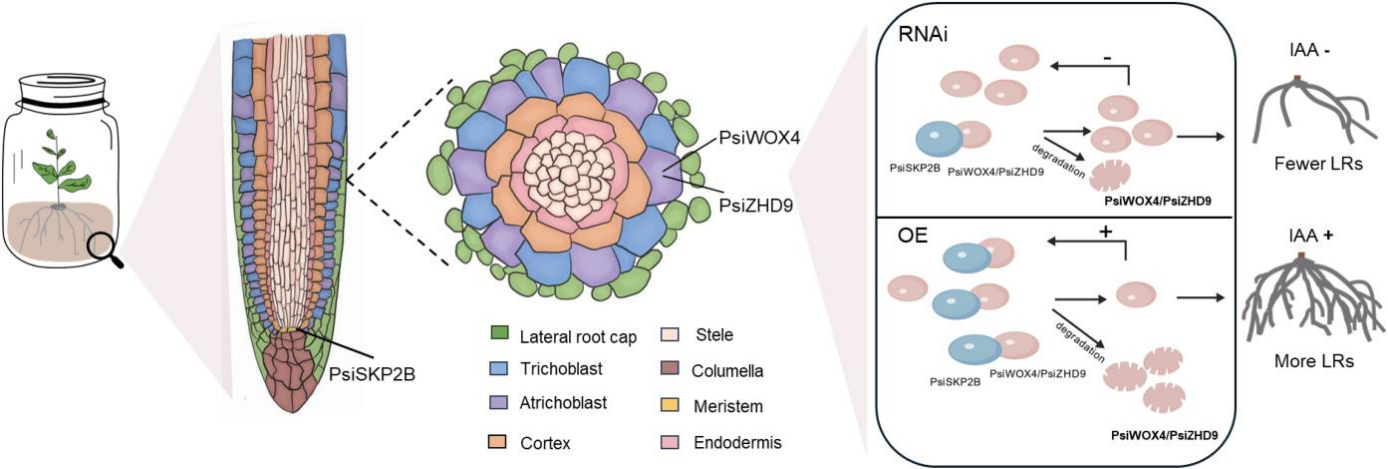
Proposed working model of the role of *PsiSKP2B* in LR development. We performed GWAS on 96 RSA traits and identified *PsiSKP2B* as a candidate gene colocalized by 6 traits. By integrating the findings from GWAS, transcriptome, and scRNA-Seq, we identified *PsiSKP2B* as a key regulator of meristematic tissue cells involved in LR development. PsiSKP2B, regulates LR development by recognizing and binding to PsiWOX4 and PsiZHD9, mediating their ubiquitination and proteasomal degradation. For *PsiSKP2B*-OE lines, the proteins PsiWOX4 and PsiZHD9 are extensively degraded, which in turn activates a feedback loop that increases their transcript levels, thereby promoting LR development. In contrast, the degradation of PsiWOX4 and PsiZHD9 is diminished for *PsiSKP2B*-RNAi lines, leading to a weakened feedback mechanism and consequently reduced transcript levels, which in turn inhibits LR development. *PsiSKP2B* is known to be expressed in meristematic cell clusters, whereas *PsiWOX4* and *PsiZHD9* are expressed in the atrichoblast, and both are enriched for auxin. This spatial arrangement provides a basis for *PsiSKP2B* to modulate *PsiZHD9/PsiWOX4* via the auxin signaling pathway, thereby influencing LR development. The “+” indicates promotion, and the “−” indicates inhibition.

*AtSKP2* serves as a component of the SCF ubiquitin ligase complex, which functions as an E3 ubiquitin ligase. This complex is responsible for recognizing and tagging target proteins for ubiquitination, marking them for degradation via the proteasome pathway. *AtSKP2* interacts with *Skp1* via its F-box structural domain and recognizes specific substrate proteins through other structural domains (LRR, WD40). The role of SCF complexes in root development, particularly in LR formation, has been extensively studied (Xu et al. 2002; Chapman and Estelle 2009; Yu et al. 2015). For example, F-box genes, such as TRANSPORT INHIBITOR RESPONSE 1 (*TIR1*), play a crucial role in auxin signaling and root development (Chapman and Estelle 2009; Yu et al. 2015). Our findings further support the importance of SCF complexes in regulating root architecture. Our studies show that PsiSKP2B, as part of the SCF complex, regulates LR development by recognizing and binding to PsiWOX4 and PsiZHD9, mediating their ubiquitination and proteasomal degradation. For *PsiSKP2B*-OE lines, the proteins PsiWOX4 and PsiZHD9 are extensively degraded, which in turn activates a feedback loop that increases their transcript levels, thereby promoting LR development. In contrast, the degradation of PsiWOX4 and PsiZHD9 is diminished for *PsiSKP2B*-RNAi lines, leading to a weakened feedback mechanism and consequently reduced transcript levels, which in turn inhibits LR development. The direct interaction between PsiSKP2B and PsiWOX4 or PsiZHD9 was further validated through Y2H experiments, corroborating this regulatory model (Fig. 6). Single-cell transcriptomic analysis revealed that *PsiSKP2B* is predominantly expressed in meristematic cells, whereas *PsiWOX4* and *PsiZHD9* are primarily expressed in atrichoblast cells (Fig. 5E; Supplementary Fig. S13; Supplementary Fig. S15, A and B). Additionally, the enrichment of IAA-related genes in meristematic and atrichoblast cells suggests that auxin signaling may play a substantial role in *PsiSKP2B*-mediated LR development (Supplementary Fig. S9, C and F). In conclusion, PsiSKP2B plays a crucial role in LR development by modulating the protein stability of PsiWOX4 and PsiZHD9, a process that may be regulated by the auxin signaling pathway.

*WOX4* is a vital member of the WUSCHEL-RELATED HOMEOBOX gene family that plays a central role in plant root development (Rasheed et al. 2024). As a key factor in stem cell regulation, *WOX4* maintains root meristem activity and promotes LR development by modulating the balance between cell division and differentiation (Sarkar et al. 2007 ). It has been shown that WOX4 interacts with other regulators (CLE peptide and WUSCHEL-RELATED HOMEOBOX 5 [WOX5]) to form a complex regulatory network that ensures precise and plastic root development (Lou et al. 2024). In *Arabidopsis* (*A. thaliana*) *WOX4* mutants, root meristem activity was substantially reduced, and LR development was inhibited, whereas *WOX4*-OE plants enhanced root growth and increased LRs (Hirakawa et al. 2010). In addition, *WOX4* regulates root development by integrating multiple hormone signaling pathways. In Japanese larch (*Larix kaempferi*), *WOX4* is involved in the LR initiation and development, which will be regulated through the IAA, JA, and ABA signaling pathways (Wang et al. 2020). Functionally, JA promotes quiescent center cell division for stem cell activation and regeneration, and IAA regulates LR formation by modulating JA homeostasis in *Arabidopsis* (Cai et al. 2014), while low concentrations of ABA promote LR formation (Zhang et al. 2023). These results suggest that the *PsiSKP2B*-*PsiWOX4* module might be involved in the hormonal signaling pathways to enhance LR development. Supporting this hypothesis, the post-induction expression of putatively orthologous genes of *PsiSKP2B* and *AtSKP2B* upregulates IAA-related genes in *A. thaliana* (Manzano et al. 2012), indicating that the *PsiSKP2B*-*PsiWOX4* module likely activates the IAA pathway. This is further supported by the predominant expression of IAA-related genes among marker genes in both meristematic and atrichoblast cells (Supplementary Fig. S9, C and F). Additionally, *PsiSKP2B* is predominantly expressed in meristematic cells, whereas *PsiWOX4* is mainly expressed in atrichoblast cells (Fig. 5E; Supplementary Fig. S15, A and B), suggesting that *PsiSKP2B* may regulate *PsiWOX4* expression through auxin signaling to control LR development.

The *ZHD9* gene belongs to the zinc finger homology domain (ZF-HD) gene family (Khatun et al. 2017). Studies have shown that *ZHD9* plays an important role in root development and response to adversity (Khatun et al. 2017). For example, ZINC FINGER HOMEODOMAIN 1 (*ZHD1*) in sunflower is upregulated under drought, salt stress, and ABA-induced expression, and its overexpression enhances plant tolerance to drought stress (Zhang et al. 2025). In soybean (*Glycine max*), overexpression of *GmZHD9* altered root architecture and promoted root development (Rizwan et al. 2025). These results suggest that *ZHD9* has conserved functions in regulating root development and response to adversity. In *Populus*, the regulatory mechanism of *PsiSKP2B* on *PsiZHD9* further reveals its role in LR development (Fig. 5C). Transcript levels of *PsiZHD9* were significantly increased in *PsiSKP2B*-OE lines, whereas *PsiZHD9* expression was significantly repressed in *PsiSKP2B*-RNAi lines (*P* < 0.001, Tukey test; Fig. 5C). The Y2H assay confirmed the direct interaction between PsiSKP2B and PsiZHD9, supporting their functional connection within the regulatory network (Fig. 5G). Additionally, single-cell transcriptomic analysis revealed that *PsiSKP2B* is predominantly expressed in meristematic cells, while *PsiZHD9* is mainly expressed in atrichoblast cells. This suggests that *PsiSKP2B* may regulate the expression of *PsiZHD9* through auxin signaling, thereby modulating LR development.

Based on the ability of PsiSKP2B to ubiquitinate and degrade PsiZHD9/PsiWOX4 and its association with auxin, we propose that auxin is involved in this degradation process. Analogous to other studies, it has been demonstrated that auxin drives LR initiation by synchronously activating the mitogen-activated protein kinase 14 (MPK14) and the MOS4-ASSOCIATED COMPLEX 3A/3B (MAC3A/3B) ubiquitin ligase to collaboratively degrade ETHYLENE-RESPONSIVE ELEMENT BINDING FACTOR 13 (ERF13). This mechanism exemplifies how auxin signaling activates specific ubiquitin ligases to eliminate proteins that inhibit LR formation (Yu et al. 2024). For *PsiSKP2B*, it is possible that auxin signaling activates the ubiquitin ligase activity of PsiSKP2B or that auxin modulates the molecular mechanisms underlying the interaction between PsiSKP2B and PsiZHD9/PsiWOX4, thereby enabling PsiSKP2B to more efficiently ubiquitinate and degrade these proteins. In *Arabidopsi*s, *SKP2B* negatively regulates LR formation by inhibiting cell division in meristematic and founder cells. By contrast, PsiSKP2B may promote LR growth by degrading PsiZHD9/PsiWOX4, thereby alleviating the inhibition of cell division or developmental processes associated with LR growth (Manzano et al. 2012).

### Quantification of RSA by multiangle image analysis using RiaRoot

The investigation of RSA presents substantial challenges, particularly in imaging root systems and identifying pertinent quantitative phenotypes from their intricate topologies (Spalding 2009 ). In this study, 3D multiangle imaging and digital phenotyping techniques, specifically RiaRoot, were utilized to characterize the morphological traits associated with RSA in Simon poplar (*P. simonii*) (Fig. 1C). Traditional methods for assessing root morphology have predominantly relied on single-angle imaging techniques. For example, the micro-canal method provides narrowly focused, localized data confined to the root canal wall (Jeudy et al. 2016). In contrast, 3D phenotyping technology promises to offer a holistic perspective on RSA by enabling the monitoring of root growth and development across both time and spatial dimensions (Mairhofer et al. 2012).

Nondestructive methods such as hydroponics and gel culture are available, yet these artificial culture systems have inherent limitations (Ozsoy et al. 2009). For example, roots in hydroponic systems tend to grow in a predominantly vertical orientation, lacking the lateral extensibility characteristic of soil-based root systems. Conventional methodologies, typically developed for model plants, generate root images with high clarity and strong background contrast (Seidenthal et al. 2022). Consequently, these approaches are not fully suitable for studying the root systems of cutting-propagated *P. simonii*. In this study, we observed variability in the germination timing of LRs in *P. simonii*. To mitigate the effects of age and positional variations, identical root segments were excised for experimental analysis. Additionally, we utilized RiaRoot software to quantify 96 distinct architectural parameters, providing a comprehensive insight into the root system dynamics of *P. simonii*.

Recent advances in automated analysis tools have facilitated the extraction of descriptors and shape descriptors from root images (Galkovskyi et al. 2012). However, comprehensive validation procedures for these tools frequently remain inadequate. In this study, the RiaRoot tool, which is built upon well-established and peer-reviewed algorithms, was employed. By leveraging the random forest algorithm, RiaRoot is trained using a synthetic library, thereby improving the accuracy of root system feature estimations. Furthermore, the revelation of 96 root microphenotypes through such an approach has enhanced the robustness of our findings, making them more reliable than those obtained from traditional methodologies for quantifying root architecture.

### GWAS, transcriptomics, and scRNA-Seq analyses enable dissection of the genetic basis of RSA in *Populus*

Recently, integrating transcriptome with GWAS has emerged as a rapid and efficient strategy for identifying candidate genes regulating RSA (Guo et al. 2020). In this study, we identified 5 key genes, including *PsiSKP2B*, *PsiORC3*, *PsiPGGT-I*, *PsiJAR1*, and *PsiLON2,* which exhibited significant associations with traits such as root geometry, spatial distribution, branching patterns, growth rate, and root area distribution (*P* ≤ 1.56 × 10^−6^ [1/*n*], Bonferroni test; Fig. 2, A to I). Through the integration of GWAS and transcriptomics analyses, *PsiSKP2B* was pinpointed as a particularly promising candidate gene involved in regulating LR development. Additionally, 150 SNPs exhibited significant effects when mapped as composite traits, indicating their potential as targets for cloning RSA-related genes in *Populus* (*P* ≤ 1.56 × 10^−6^ [1/*n*], Bonferroni test; Supplementary Table S2). Thus, the combination of high-throughput 3D imaging, multitrait analysis, and modern sequencing methods is envisioned as a powerful strategy for bridging the phenotype–genotype gap (Fusi et al. 2022).

We have also enhanced this approach by incorporating scRNA-seq into GWAS, which elucidated the cell-type specificity of microscopic phenotypic traits within root systems. Interpreting GWAS from the perspective of scRNA-seq has the potential to deepen our understanding of the intricate molecular mechanisms driving LR development (Serrano-Ron et al. 2021). scRNA-seq data reveal that *PsiSKP2B* is highly expressed in the meristematic cells of LR, suggesting that it may promote LR development by regulating the activity of these cells, such as cell division and differentiation. Compared with the single-omics scheme, our multiomics strategy offers insights into the symbiotic regulation and function of causal genes in LR development, highlighting the coordinated regulatory characteristics of LR development.

In this study, we adopted a holistic approach by integrating GWAS, transcriptomics, scRNA-seq, and phenomics to elucidate the intricate interactions between genotype and phenotype, enabling the effective identification of candidate genes underpinning RSA. The synergy among these multiomics layers establishes a robust framework for deciphering the genetic underpinnings of the genotype–phenotype relationship, as highlighted by Wilson et al. (2015). In previous studies, multiomics investigations have yielded substantial insights into the root systems of *A. thaliana*, revealing genes critical for cell wall elongation and highlighting candidate loci integral for salt-stress-induced root growth (Kobayashi et al. 2016). Additionally, the concurrent analysis of the genome, transcriptome, and metabolome has elucidated candidate genes associated with 6 agronomic traits in maize (*Zea mays*) (Xu et al. 2017). Multiomics approaches have been crucial in identifying key membrane transporters associated with wood formation in *Populus* (Obudulu et al. 2018). However, our study represents the application of a multiomics strategy to map RSA-associated genes in a woody plant, marking an advancement in this field.

## Materials and methods

### Association population of *P. simonii*

The natural population of Simon poplar (*P. simonii*), consisting of 303 unrelated accessions, was collected from 9 Chinese provinces and cities, including Beijing, Hebei, Henan, Shanxi, Liaoning, Inner Mongolia, Gansu, Ningxia, and Qinghai, and planted in a clonal arboretum in Guan Xian County, Shandong Province, China (36°23′N, 115°47′E) (Fig. 1A). The clonal arboretum, established in 2018, was propagated asexually via root cuttings and organized according to a randomized complete block design across 3 distinct blocks. In April, branches from dormant *Populus* were harvested and subjected to hydroponic growth. All the above materials were collected with 3 biological replicates.

### 3D imaging platform

For clonal propagation, 2-cm shoot segments were excised from surface-sterilized plantlets and cultivated in large tubes filled with a transparent medium at 25 °C under a 12-h light/dark cycle with a light intensity of 2,000 l×. A specialized plant photography platform was utilized, consisting of a round acrylic cylinder with a height of 60 cm, an inner diameter of 30 cm, and an outer diameter of 32 cm, alongside an MT370 series turntable. This turntable, which connects to a personal computer (PC) and a camera (Nikon, Tokyo, Japan) via Bluetooth, facilitated the submerged photography of large tubes within the cylinder (Fig. 1C). The apparatus was programmed to automatically capture images at a 15° angle, generating 24 two-dimensional (2D) images per accession (Fig. 1D). All accessions were performed in 3 biological replicates.

### Phenotypic data of the *P. simonii* association population and correlation analysis

To estimate RSA in the *P. simonii* population, 96 microphenotype traits were measured using a custom ImageJ plugin named RiaRoot (Fig. 1B), which employs the random forest algorithm for analysis (Lobet et al. 2017). We employed multiangle image acquisition technology, combined with a self-developed image segmentation method, to preprocess the root system images and obtain “cleaned images.” These cleaned images were then analyzed using the RiaRoot software script to extract morphological and architectural characteristics of the root systems. During phenotypic feature extraction, image segmentation further delineated macroscopic traits into multiple microscopic phenotypes. For example, the macroscopic trait cross_hori_mean (mean roots detected when scanning the root system with a horizontal line at the depth) was segmented into 30 layers of microphenotypes (cross_hori_0_mean to cross_hori_29_mean). This plugin facilitated the extraction of various descriptors from the root image, including total root length, projected area, and number of visible root tips. Additionally, shape descriptors such as the convex hull area of the root system and the exploration ratio (the ratio between the root system's width and depth) were also extracted (Lobet et al. 2017). Further enhancements were made to these shape descriptors, as detailed in Supplementary Table S1. To quantify the phenotypic correlations, we employed Pearson's correlation analysis to assess linear relationships between phenotypic traits. All statistical analyses were performed using R language, with the cor() function for Pearson's correlation.

### PCA, population genetics, and LD analysis

The methods for resequencing the association population with 303 accessions have been previously described (Xiao et al. 2021). In brief, clean reads were aligned to the *P. simonii* reference genome by the Burrows–Wheeler Aligner (BWA) using default parameters (Li and Durbin 2009). Reads with low mapping quality (MQ < 20) were filtered and removed using SAMtools (v. 1.1). Genome variant calling was conducted using Genome Analysis Toolkit v. 4.0 (GATK) with conservative parameters. Following the removal of those with a MAF ≥ 0.05 and missing rate ≤ 1, we identified 639,988 SNPs across all individuals (Danecek et al. 2011; Yang and Wang 2015). An individual-based neighbor-joining (NJ) tree was constructed using MEGA7, based on the *P*-distance for the 303 accessions, with 1,000 bootstrap replications to assess robustness (Kumar et al. 2016). The population genetic structure was examined using the program fastStructure (Raj et al. 2014), with the number of ancestral populations (*K*) ranging from 2 to 4. We computed the average CV error for every *K*-value. Population structure was assessed using a kinship matrix generated via the “GRM” method in GCTA v1.93 (Yang et al. 2011). The matrix visualized pairwise genetic relationships among the 303 *P. simonii* accessions, revealing low levels of relatedness (95.78% of kinship coefficients < 0.05), indicating a diverse panel suitable for GWAS. The phylogenetic tree was divided into 3 clades, consistent with the fastStructure results. Thus, *K* = 3 was the optimal K-value for distinguishing subpopulations. PCA was conducted using the GCTA software to identify and correct for population stratification (Yang et al. 2011). The variance explained by each principal component was calculated to assess the contribution of each component to the overall genetic variation. To estimate LD in the *P. simonii* population, the squared correlation coefficient (*r*^2^) between pairwise SNPs was calculated using PopLDdecay (Zhang et al. 2019), with parameters set as “-MaxDist 1000 kb -MAF 0.05 -Miss 0.1.”

### Genome-wide association analysis

GWAS was performed in EMMAX software using high-quality data for the 639,988 SNPs (Kang et al. 2010). A standard MLM was applied, incorporating population structure (Q) and kinship (K) as covariates, with both parameters estimated as previously described. The adjust option was utilized to apply Bonferroni correction, thereby ascertaining the genome-wide significance across 96 traits, with a suggestive *P*-value ≤ 1.56 × 10^−6^ (*P* = 1/*n*; *n* = the effective number of independent SNPs) (Li et al. 2012). Genes within 2 kb upstream or downstream of the GWAS signal, or those overlapping with it, were considered candidate genes.

### RNA-seq analysis of LRs

LR samples were collected from 3 positional regions of the main root: upper (root-1 to root-3 were collected from the basal 1/3 of the main root), middle (root-4 to root-6 were collected from the central 1/3 of the main root), and lower (root-7 to root-9 were collected from the apical 1/3 of the main root, including the root tip and meristematic zone). This spatial segmentation allowed us to profile gene expression along the root axis during LR development. Leaf samples (leaf-1 to leaf-7) were collected as controls. The sampling duration was minimized to minimize changes in gene expression. Total RNA was isolated from the leaf and LR of 40-d wild-type (WT) *P. simonii* in 3 independent biological replicates using TRIzol reagent (Invitrogen). High-throughput sequencing was performed on the Illumina NovaSeq sequencing platform (Illumina, San Diego, CA), generating 100-bp paired-end reads. Each sample was also sequenced separately, but the data from all replicates were pooled during the analysis stage to increase statistical power and minimize technical variability. The clean reads were aligned to the *P. simonii* genome using HISAT2 (v.2.0.5) with default parameters (Kim et al. 2015). Gene expression levels were quantified as fragments per kilobase of transcript per million (FPKM) using featureCounts (v.2.0.2) with default parameters (Liao et al. 2014). Transcript abundances were normalized using the Z-score method (Curtis et al. 2016). Differential expression analysis was performed using the DESeq R package (Varet et al. 2016). The gene with *P*-value < 0.05 and |log2foldchange| > 1 was defined as a DEG. GO enrichment analysis of DEGs was performed using TBtools, based on the poplar reference genome annotations in the GO database. Gene co-expression modules were identified using the WGCNA package in R (Langfelder and Horvath 2008), employing hierarchical clustering analysis with parameters: mergeCutHeight = 0.25, deep split = 2, module size = 12, numericLabels = TRUE, pamRespectsDendro = FALSE, and pamStage = TRUE.

### Phylogenetic analysis and multiple sequence alignment

The SKP2 proteins were aligned using ClustalW 2.0 (Edgar 2004). The phylogenetic tree was constructed on the basis of the maximum likelihood method via MEGA 11 software, with 1,000 bootstrap replications to assess robustness (Kumar et al. 2016). Multiple sequence alignment was performed using ESPript 3.0 (Gouet et al. 1999).

### Preparation of root samples for scRNA-seq

Root tips (2 cm) were collected from primary roots at 40 d post-rooting (no LR sample, characterized by LR primordia with small protrusions) and from roots with established LR at 40 d post-rooting (LR sample). The root tips were digested for 2 h at 28 °C in an RNase-free enzyme solution containing 4% cellulase, 1.5% allicin, 0.4 m mannitol, 0.1 m 4-morpholineethanesulfonic acid, 10 m KCl, 10 m CaCl_2_, and 0.1% bovine serum albumin (BSA). After digestion, the samples were briefly centrifuged to separate protoplasts from cellular debris. Protoplasts were then resuspended in an appropriate concentration of mannitol solution. A hematocrit plate was employed to count the protoplasts, assess the extent of cell wall breakage, and determine the fragmentation rate. Protoplast viability was determined using Taipan Blue staining (Zhang et al. 2023), with a viability ratio exceeding 85%. This high viability rate ensured that the majority of cells were intact and suitable for downstream scRNA-seq analysis. The protoplast concentration was adjusted to 150 to 200 cells/μL. Finally, the qualified protoplasts were encapsulated into droplets according to the 10× Genomics Single Cell Protocol (Becht et al. 2019). The sequencing depth per cell was also carefully monitored, with an average read depth of 86,460 reads per cell. This depth ensures sufficient coverage for accurate gene expression quantification.

### scRNA-seq library construction and sequencing

The libraries were constructed using Chromium Controller and Chromium Single Cell 30 Reagent Kits v2. Briefly, cell suspensions in a chip were loaded on a Chromium Controller (10× Genomics, Pleasanton, CA) to generate single-cell GEMs (gel beads in emulsion). The scRNA-seq library was then prepared using the Chromium Single Cell 30 Gel Bead and Library Kit (P/N #120236, 120237, 120262; 10× Genomics). Library quality was assessed using an Agilent 2100 Bioanalyzer (Agilent Technologies, Santa Clara, CA), and the DNA concentration was quantified using a Qubit (Thermo Fisher). Sequencing was performed on an Illumina NovaSeq platform (Berry Genomics).

### Preprocessing of raw scRNA-seq data and cell clustering

The raw scRNA-seq data were first analyzed by Cell Ranger 2.2.0. These pipelines aligned sequencing reads to a reference genome and generated gene-cell matrices (Satija et al. 2015). Raw UMI counts were log-normalized using the LogNormalize method in Seurat (scale factor = 10,000). The raw scRNA-seq reads from root tissues of 2 distinct conditions (no LR sample and LR sample) were mapped to the *P. simonii* reference genome, using the “cellranger count” function in Cell Ranger 2.2.0. The gene-cell matrices (named “filtered_gene_bc_matrices” by 10× Genomics), generated using Cell Ranger 2.2.0, were utilized as the raw dataset for subsequent analyses. Initial quality filtering removed genes expressed were fewer than 3 cells. Potential doublets were identified and removed using scDblFinder v1.15.4 (Germain et al. 2021). The average doublet rate across samples only was 7.12%. To assess the conservation across the 2 root developmental stages, scRNA-seq data were integrated and analyzed jointly using the SCTransform-based operation, which employs a generalized linear model (GLM) to normalize data across samples, effectively eliminating technical noise and biological variation (Hafemeister and Satija 2019). Integration was achieved through canonical correction analysis (CCA) in the Seurat package (v3.1.0), which used an anchor-based method to correct for batch effect (Satija et al. 2015). Dimensionality reduction was conducted via PCA, selecting the top 30 PCs for unsupervised clustering. Within the resolution spectrum of 0.4 to 1.6, cell clusters were visualized using nonlinear dimensional reduction algorithms (“RunTSNE” and “RunUMAP”). The highly variable genes (HVGs) were selected based on variance-stabilizing transformation (VST) using the SCTransform algorithm for downstream analysis (Hafemeister and Satija 2019). Cluster marker genes were identified using Seurat function “FindAllMarkers.” The cluster-enriched genes were identified by parameters of “logfc.threshold = 0.58,” indicating that the log_2_ fold change of average expression is more than 0.58. The cell type annotation of each cluster was determined manually, referencing both homologous genes from *Arabidopsis* (*A. thaliana*) and genes enriched within each cluster. The repeated analysis has been conducted multiple times, utilizing the same or varying parameters.

### Vector construction and genetic transformation of *Populus*

To generate the transgenic plants, the full-length CDSs of *PsiSKP2B* without the stop codon were amplified from *P. simonii* using primer pairs described in Supplementary Table S19. The CDS was cloned into the pBI121 vector at the *Xba*I site driven by the CaMV 35S promoter and introduced into *Agrobacterium tumefaciens* strain GV3101. The *PsiSKP2B*-RNAi construct was constructed by inserting a fragment of *PsiSKP2B* with a partial inverted repeat into the pEGOEP35S-H vector, behind the CaMV 35S promoter. Both overexpression and RNAi vectors were transformed into the 84K poplar (*Populus alba* × *Populus glandulosa*) via the *Agrobacterium*-mediated method (Song et al. 2021). Positive transgenic lines were confirmed with a forward primer derived from ∼20 bp of the CaMV 35S promoter and a reverse primer derived from ∼20 bp of *PsiSKP2B*. Detailed information on all primers used for constructing the vectors is provided in Supplementary Table S19.

### RNA-seq analysis of transgenic lines

The expression of *PsiSKP2B* in transgenic roots was determined by RT-qPCR 11 d after 84K poplars were transformed. On roots from control, overexpressed (OE-1 and OE-9), and silenced *PsiSKP2B* (Ri-1 and Ri-2) transgenic lines, transcriptome sequencing was performed. The same procedure as described in RNA-seq analysis of LRs. Clean reads obtained from raw data were mapped to *P. simonii* genome using HISAT2 (Kim et al. 2015). Gene expression levels were quantified as FPKM using featureCounts (v.2.0.2) with default parameters (Liao et al. 2014). Differential expression analysis was performed using the DESeq R package (Varet et al. 2016). The gene with *P*-value < 0.05 and |log2foldchange| > 1 was defined as a DEG. GO enrichment analysis of DEGs was conducted using TBtools, based on annotations from the poplar reference genome in the GO database.

### Subcellular localization

The ORFs of *PsiSKP2B*, *PsiWOX4*, and *PsiZHD9* (without stop codons) were cloned into the pBI121 vector with *Bax*I restriction sites and transformed into *A. tumefaciens*. The *Agrobacterium* cells were harvested and suspended in the resuspending solution (10 mm MgCl_2_, 10 mm MES, and 200 μM acetosyringone). After 2 to 4 h at 28 °C, the suspensions were infiltrated into *N. benthamiana* leaves (He et al. 2018). Three days after injection, the fluorescence signals were visualized using the confocal laser scanning microscope (enhanced GFP [eGFP], 488 and 510 nm; mCherry, 561 and 610 nm) (TCS SP8; Leica). The primers used are listed in Supplementary Table S19.

### Y2H assay

Y2H assay was performed using the Matchmaker Gold Y2H Library Screening System according to the manufacturer's instructions (OEbiotech, China). The *PsiSKP2B* was fused into the pGBKT7 vector, and *PsiWOX4* and *PsiZHD9* were fused into the pGADT7 vector, both using *Eco*RI restriction sites. PsiSKP2B and PsiWOX4 or PsiZHD9 proteins were co-transformed into yeast strains Y2H Gold, respectively, and the resulting colonies were tested on selective medium (SD/-Leu/-Trp) and then on quadruple dropout medium (SD/-Ade/-His/-Leu/-Trp/400 ng/mL AbA) for 5 d at 30 °C. The vectors pGBKT7-53 and pGADT7-T served as the positive control, whereas pGBKT7-lam and pGADT7-T were employed as the negative control. The primers used are listed in Supplementary Table S19.

### RT-qPCR

Total RNA was extracted from plant root samples using TRIzol (Invitrogen) and subsequently reverse-transcribed into cDNA using PrimeScript RT kit (Takara, Japan). RT-qPCR was performed using ChamQ Universal SYBR qPCR master mix (Vazyme, Q711-02), with poplar *Ubiquitin* gene as the reference gene. Quantitative assessment of gene expression relied on the comparative 2^−ΔΔCt^ method (Livak and Schmittgen 2001). The primers used are listed in Supplementary Table S19. All RT-qPCR assays were performed in 3 biological replicates and 3 technical replicates.

### Split-luc complementation assays

The full-length ORFs of *PsiWOX4* and *PsiZHD9* were cloned into pCAMBIA1300-Nluc vector with *Kpn*I and *Sal*I restriction sites, respectively. The full-length ORFs of *PsiSKP2B* were cloned into pCAMBIA1300-Cluc vector with *Kpn*I and *Sal*I restriction sites. The primers used are listed in Supplementary Table S19. The constructs were introduced into *Agrobacterium* strain GV3101, and plasmids of different combinations were transiently co-infiltrated into *N. benthamiana* leaves. After 48 h of infiltration, D-luciferin potassium salt (150 µg/mL) was sprayed onto the leaves of *N. benthamiana*. The leaves were harvested and kept in the dark for 5 min before determining luminescence signals by a CCD camera (TANAON 5200, Shanghai).

### Co-IP assays

For co-IP assays, the CDSs of *PsiSKP2B*, *PsiWOX4*, and *PsiZHD9* were amplified and inserted into the pBI121-GFP and pCAMBIA1300-FLAG vectors, respectively. The specific primers used are listed in Supplementary Table S19. Mixtures of *A. tumefaciens* containing *PsiZHD9*-Flag/*PsiWOX4*-Flag or Flag empty vector and *PsiSKP2B*-GFP were infiltrated into *N. benthamiana* leaves for 72 h. Total proteins were extracted with IP buffer (50 mm Tris-HCl [pH 7.4], 150 mm NaCl, 1 mm ethylenediaminetetraacetic acid [EDTA], 1% Triton X-100, and 1% protease inhibitor cocktail [Bimake]) and then immunoprecipitated with DYKDDDDK-Nanoab-Magnetic beads (FNM-25-1000) according to the manufacturer's instructions. Immunoprecipitated proteins were separated by SDS–PAGE (4% to 25% gel) and detected by immunoblotting with anti-GFP (TRAN, HT801-01, diluted 1:5,000) or anti-Flag antibody (Sigma, F1804, diluted 1:5,000). After incubation with a secondary antibody (TRAN, HS201-01, diluted 1:5,000) for 1 h, the immunoblot signal was visualized with Super ECL (ACE; BK0042-01).

### In vivo ubiquitination assays

To identify PsiSKP2B-mediated ubiquitination of PsiWOX4 and PsiZHD9, *PsiZHD9*-Flag or *PsiWOX4*-Flag was expressed alone or co-expressed with *PsiSKP2B*-GFP in *N. benthamiana* leaves for 4 h in the presence of 50 μM MG132 or DMSO. The *PsiWOX4*-Flag or *PsiZHD9*-Flag and GFP combination was used as a negative control. Total proteins were extracted and immunoblotted with the anti-GFP (TRAN, HT801-01, diluted 1:5,000) and anti-Flag antibody (Sigma, F1804, diluted 1:5,000) to ensure protein expression. After incubation with a secondary antibody (TRAN, HS201-01, diluted 1:5,000) for 1 h, the immunoblot signal was visualized with Super ECL (ACE; BK0042-01).

### Enzyme-linked immunosorbent assay

Fresh plant tissues (roots) were harvested and immediately frozen in liquid nitrogen. Tissues (0.1 to 0.5 g) were ground to a fine powder using a mortar and pestle under liquid nitrogen. The powder was transferred to a 2-mL centrifuge tube, and 1 mL of ice-cold extraction buffer (80% methanol containing 1 mm butylated hydroxytoluene and 0.1% acetic acid) was added. Tubes were vortexed vigorously and incubated at 4 °C with shaking for 16 to 20 h to extract endogenous IAA. Samples were centrifuged at 16,000 × *g* for 20 min at 4 °C, and the supernatant was transferred to a new tube. The pellet was re-extracted with 500 μL of extraction buffer, and the combined supernatants were dried under a stream of nitrogen at 30 °C. The residue was reconstituted in 500 μL of phosphate-buffered saline (PBS, pH 7.4) containing 0.1% Tween-20 (PBST) and filtered through a 0.22-μm syringe filter to remove particulates. A commercial IAA ELISA kit (MyBioSource, USA) was used according to the manufacturer's instructions. To quantify IAA levels, a microtiter plate was coated with purified IAA-specific monoclonal antibody to create a solid-phase antibody. Sequentially, IAA standards or samples were added to the antibody-coated wells, followed by the addition of horseradish peroxidase (HRP)-conjugated IAA antibody. This process forms an antibody–antigen–enzyme-labeled antibody complex. After thorough washing to remove unbound components, the substrate 3,3′,5,5′-tetramethylbenzidine (TMB) was added to the wells. The HRP enzyme catalyzes the conversion of TMB to a blue color, which then changes to yellow in the presence of acid. The intensity of the yellow color is directly proportional to the concentration of IAA in the sample. Absorbance (optical density, OD) was measured at 450 nm using a microplate reader, and the IAA concentration in the samples was determined by interpolation from a standard curve.

### RNA in situ hybridization

The specific regions of the marker genes were amplified and cloned into the pGEM-TEasy vector (Promega). Subsequently, in vitro transcription and labeling were performed using the Digoxigenin RNA Labeling Kit (Roche). The hybridization and immunological detection procedures were carried out as previously described (Zhao et al. 2009; Liang et al. 2015). Microscopy was conducted using a Leica DM6 B microscope in bright-field mode. The primers used for these analyses are listed in Supplementary Table S19.

### Statistical analysis

Statistical significance was assessed using GraphPad Prism 10 software, employing 1-way ANOVA coupled with Tukey's multiple-comparison test, with *P* < 0.05 denoting significance. Significant differences were indicated by *P < 0.05, **P < 0.01, ***P < 0.001, or ****P < 0.0001, ns, with no significant difference. The data were presented as mean ± SEM from at least 3 biological replicates.

### Accession numbers

Sequence data from this article can be found in the GenBank/EMBL data libraries under accession numbers: *PsiSKP2B* (*Psi05G016630*), *PsiJAR1* (*Psi02G016690*), *PsiLON2* (*Psi01G014380*), *PsiORC3* (*Psi13G007600*), *PsiPGGT-I* (*Psi10G011730*), *AtSKP2* (*AT1G77000*), *SKP2A* (*AT1G21410*), *SKP2B* (*AT1G77000*), *PsiWOX4* (*Psi02G012270*), *PsiZHD9* (*Psi04G011440*), *PsiSCYL2B* (*Psi03G021500*), *PsiSRO4* (*Psi12G006720*), *PsiPNP-A* (*Psi03G009820*), *PsiRID1* (*Psi18G007370*), *PsiSAUR71* (*Psi02G005730*), *PsiCOPT5* (*Psi06G020530*), *PsiBG1* (*Psi16G005400*).

## Supplementary Material

kiaf432_Supplementary_Data

## Data Availability

The GWAS resequencing, RNA-seq data, and scRNA-seq data in this study have been deposited in the Genome Sequence Archive in the National Genomics Data Center, Beijing Institute of Genomics (China National Center for Bioinformation) of the Chinese Academy of Sciences, with accession number GVM001088 (https://bigd.big.ac.cn/gvm/getProjectDetail?Project=GVM001088), CRA026966 (https://ngdc.cncb.ac.cn/gsa/s/gJ1iTJNx), CRA026969 (https://ngdc.cncb.ac.cn/gsa/s/3WBTo9rr), and CRA012587 (https://ngdc.cncb.ac.cn/gsa/search?searchTerm=CRA012587).

## Supplementary data

The following materials are available in the online version of this article.

**Supplementary Figure S1.** Phenotypic correlation of 6 macrophenotypes in *P. simonii* natural population.

**Supplementary Figure S2.** Population structure of natural population of *P. simonii*.

**Supplementary Figure S3.** Kinship analysis of 303 *P. simonii* accessions.

**Supplementary Figure S4.** Domain analysis and sequence alignment of *PsiSKP2B*.

**Supplementary Figure S5.** Clustering dendrogram of genes and RT-qPCR of candidate genes.

**Supplementary Figure S6.** Sequence identity of SKP2 proteins.

**Supplementary Figure S7.** The relationship between clusters at different resolutions and visualization.

**Supplementary Figure S8.** Single-cell RNA-seq and cluster annotation of *Populus* root tips.

**Supplementary Figure S9.** Cell-type-specific genetic and functional annotations.

**Supplementary Figure S10.** Expression analysis of *PsiSKP2B* in transgenic plants.

**Supplementary Figure S11.** IAA concentration of *PsiSKP2B* transgenic and WT plants.

**Supplementary Figure S12.** GO enrichment of the DEGs in *PsiSKP2B* transgenic plant.

**Supplementary Figure S13.** The comparison of 6 genes’ (*PsiSKP2B*, *PsiWOX4*, *PsiSAUR71*, *PsiZHD9*, *PsiCOPT5*, and *PsiBG1*) expression between different cell types.

**Supplementary Figure S14.** PsiSKP2B ubiquitinates and targets PsiWOX4 and PsiZHD9 for degradation.

**Supplementary Figure S15.** RNA in situ hybridization detection and UMAP visualization of cell-type-specific marker genes.

**Supplementary Table S1.** Description of root system traits.

**Supplementary Table S2.** Full details of GWASs.

**Supplementary Table S3.** Go annotation results of GWAS-related gene sets.

**Supplementary Table S4.** LR and leaf transcriptome alignment to the *P. simonii* genome.

**Supplementary Table S5.** DEGs in the LRs of *P. simonii*.

**Supplementary Table S6.** Functional enrichment of DEGs in the LRs of *P. simonii*.

**Supplementary Table S7.** Genes of co-expression network modules.

**Supplementary Table S8.** Single-cell transcriptomes of LR and no LR samples alignment to the *P. simonii* genome.

**Supplementary Table S9.** Expression of marker gene specifically in each cluster.

**Supplementary Table S10.** Identification of cell type in *Populus* root.

**Supplementary Table S11.** Functions of marker genes in each cell type.

**Supplementary Table S12.** Proportion of cell numbers in each cell type in LR and no LR samples.

**Supplementary Table S13.** Marker genes per cell type for LR and no LR samples.

**Supplementary Table S14.** WT lines and OE lines DEGs.

**Supplementary Table S15.** WT lines and RNAi lines DEGs.

**Supplementary Table S16.** Functional enrichment of WT and transgenic lines DEGs.

**Supplementary Table S17.** Gene co-expression network of the turquoise module.

**Supplementary Table S18.** Gene name with weight values in the top 20 in turquoise module.

**Supplementary Table S19.** List of primers used for vector construction.

## Dive Curated Terms

The following phenotypic, genotypic, and functional terms are of significance to the work described in this paper:

SKP2A Gramene: AT1G21410
SKP2A Araport: AT1G21410

PsiSKP2B Gramene: AT1G77000
PsiSKP2B Araport: AT1G77000
