# Supplementary material for: Integrating 3D imaging, GWAS, and single-cell transcriptome approaches to elucidate root system architecture in Populus
Source: Plant Physiol. 2025 Sep 26;199(2):kiaf432. doi: 10.1093/plphys/kiaf432 (PMC12526957; doi:10.1093/plphys/kiaf432)
Supplement: kiaf432_Supplementary_Data [file kiaf432_supplementary_data.zip › Supplementary Data.pdf]

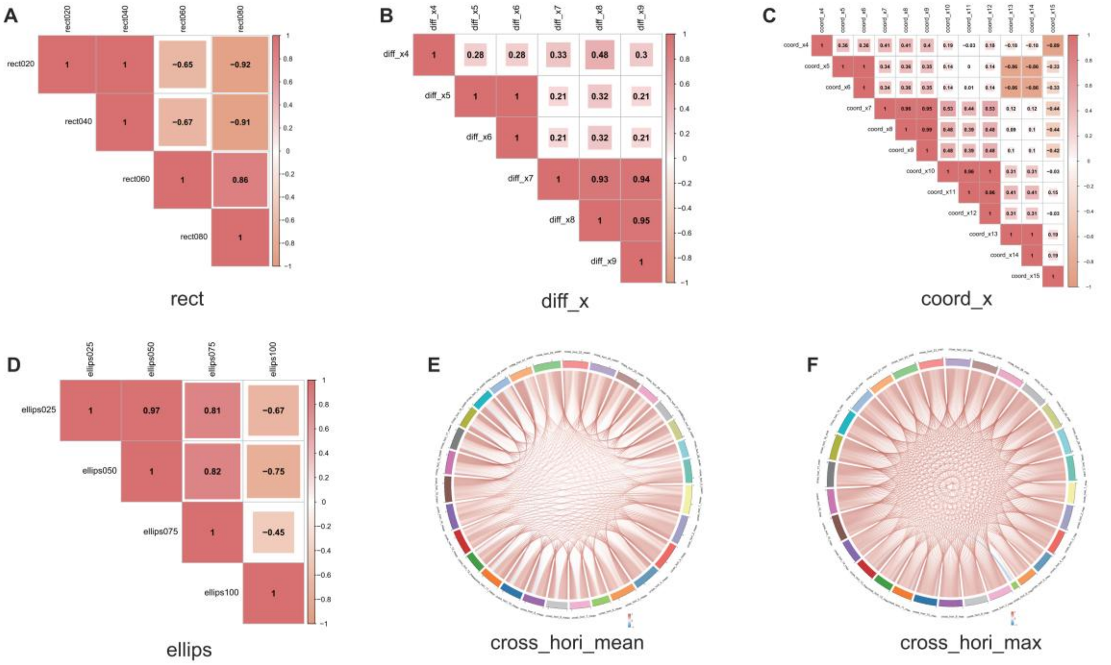

Supplementary Figure S1. Phenotypic correlation of six macrophenotypes in *Populus simonii* natural population. Phenotypic correlation of macrophenotypes (rect **(A)**, diff_x **(B)**, coord_x **(C)**, ellips **(D)**, cross_hori_mean **(E)**, cross_hori_max **(F)**) in *P. simonii* natural population. **A** to **D)** Orange indicates negative correlations and red indicates positive correlations. The width of colored box corresponds to the absolute value of the displayed number and represents correlation strength. **E** to **F)** Blue indicates negative correlations and red indicates positive correlations. The thickness and color of the ribbons correlate to the correlation of phenotypes.

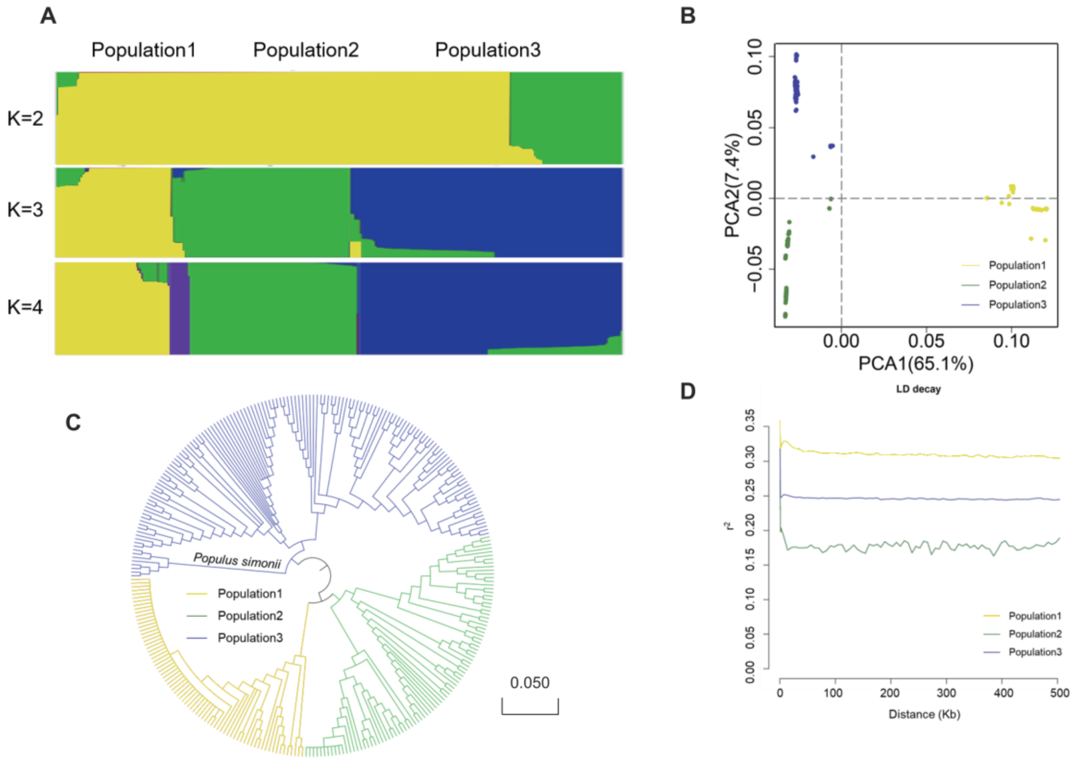

Supplementary Figure S2. Population structure of natural population of *P. simonii*. A) Population structure of the 303 *P. simonii* accessions at each value of K ranging from 2 to 4. The color of the lines indicates different groups of *P. simonii* accessions. Yellow represents Population 1. Green represents Population 2. Blue represents Population 3. **B)** Principal component analysis (PCA) plots of the first two components of *P. simonii* accessions. The color of the lines indicates different groups of *P. simonii* accessions. **C)** The phylogenetic tree of 303 accessions. The color of the lines indicates different groups of *P. simonii* accessions. **D)** Subpopulation-specific linkage disequilibrium (LD) decay for three subpopulations.

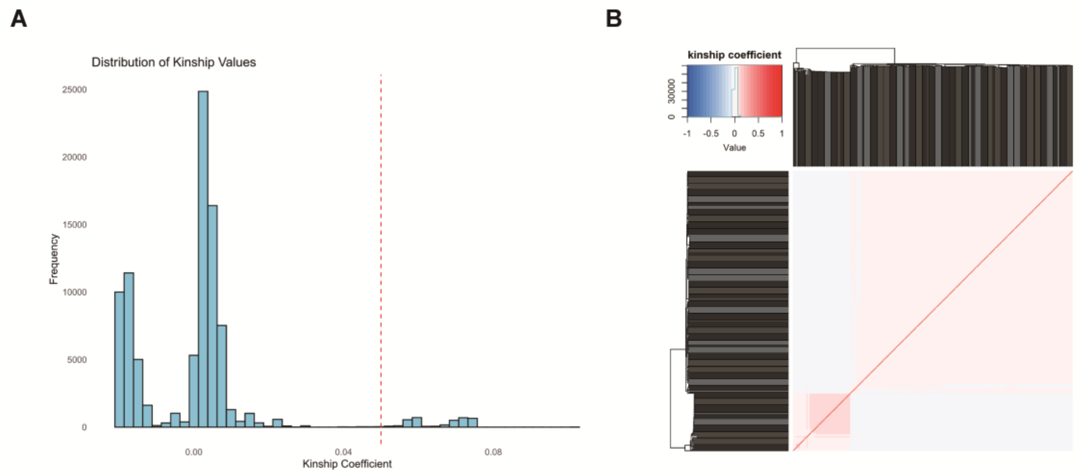

Supplementary Figure S3. Kinship analysis of 303 *P. simonii* accessions. A) Histogram showing the kinship status of *P. simonii* accessions. The horizontal axis represents the kinship coefficient. The vertical axis represents the number of sample pairs. The red dashed line represents the threshold Kinship = 0.05. **B)** Kinship matrix showing the relatedness of *P. simonii* accessions. Colors indicate kinship coefficient between accessions. Blue represents low kinship. Red represents high kinship. The diagonal elements of the kinship matrix equal 1, indicating each individual's closest relationship is with itself. Off-diagonal elements represent pairwise relatedness, where values approaching 1 signify closer genetic relationships.

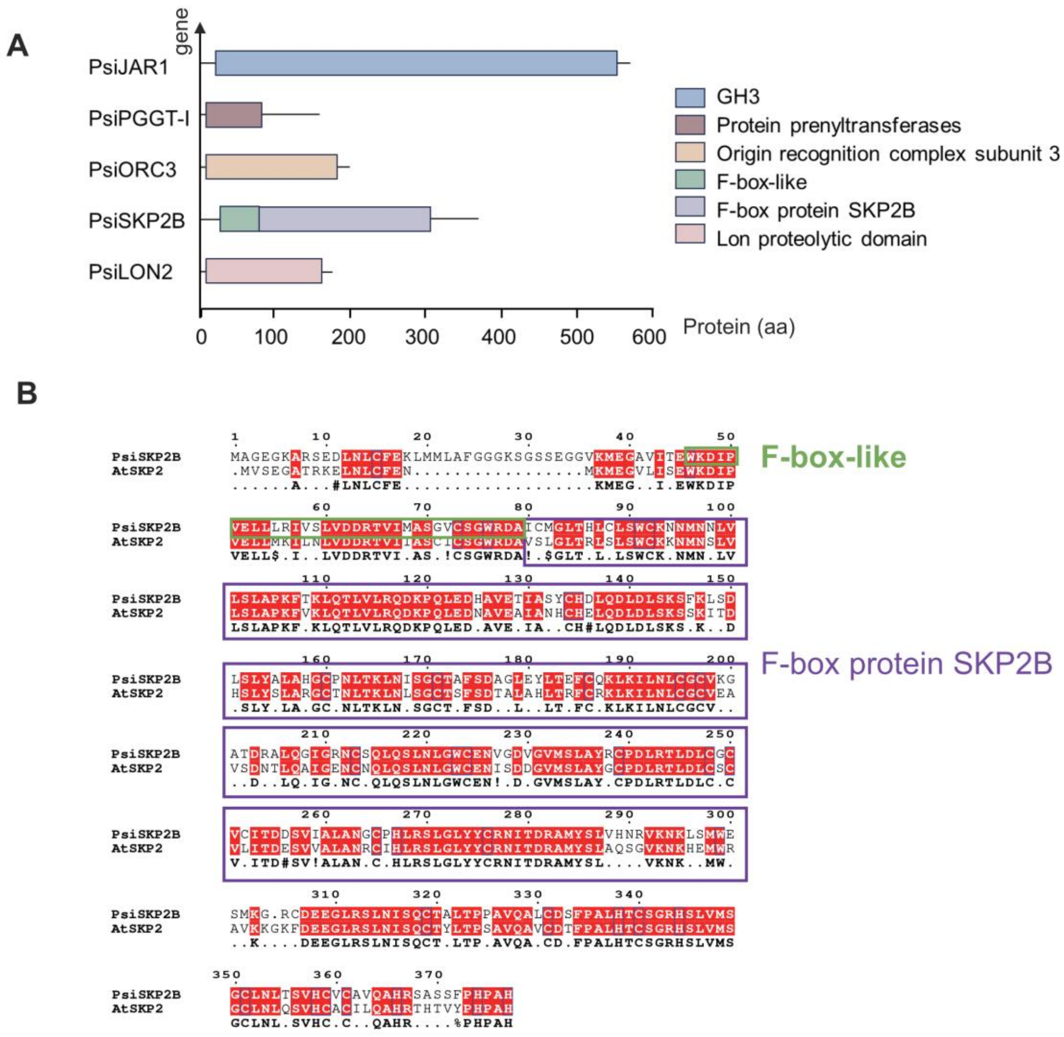

Supplementary Figure S4. Domain analysis and sequence alignment of PsiSKP2B. A) Domain analysis of candidate genes. Various domains are highlighted with color-coded. **B)** Alignment of the amino acid sequences of SKP2B from *P. simonii* and *A. thaliana*.

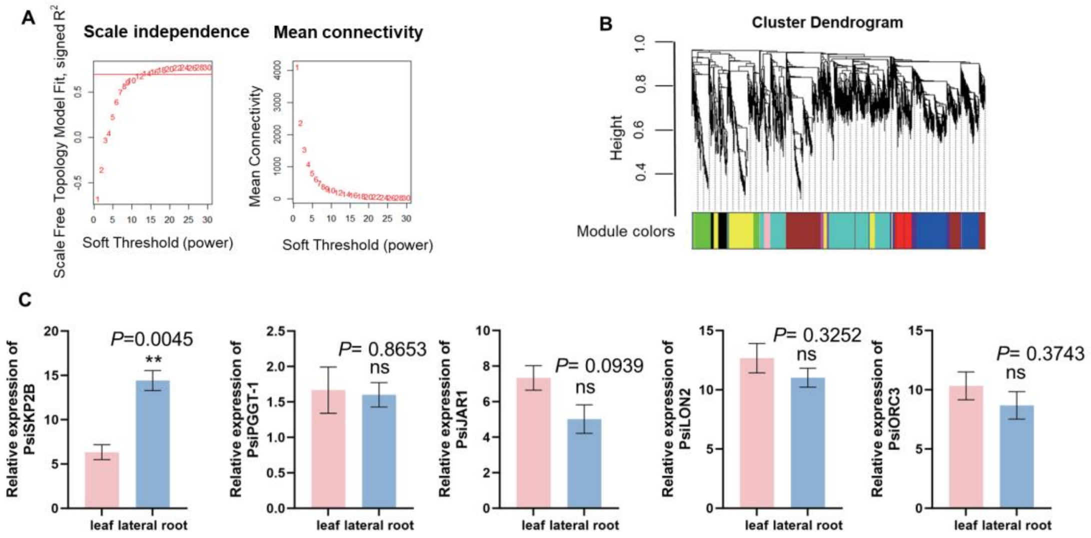

Supplementary Figure S5. Clustering dendrogram of genes and RT-qPCR of candidate genes. A) Soft threshold power estimation in the Weighted gene co-expression network analysis (WGCNA). **B)** Hierarchical cluster tree shows co-expression modules identified via the Dynamic Tree Cut method. Different modules are marked by different colors. Each leaf on the tree represents a single gene. The primary branches of the tree are segmented into twelve distinct modules, each labelled with a unique color. **C)** Expression levels of *PsiSKP2B*, *PsiPGGT-1*, *PsiJAR1*, *PsiLON2*, *PsiORC3* in leaf and lateral root, as determined by RT-qPCR. Data are shown as mean ± SEM with three biological replicates. Different asterisks (*) numbers indicate statistically significant differences (one-way ANOVA followed by post hoc Tukey test; * for $P < 0.05$, ** for $P < 0.01$, and *** for $P < 0.001$); ns, not significant.

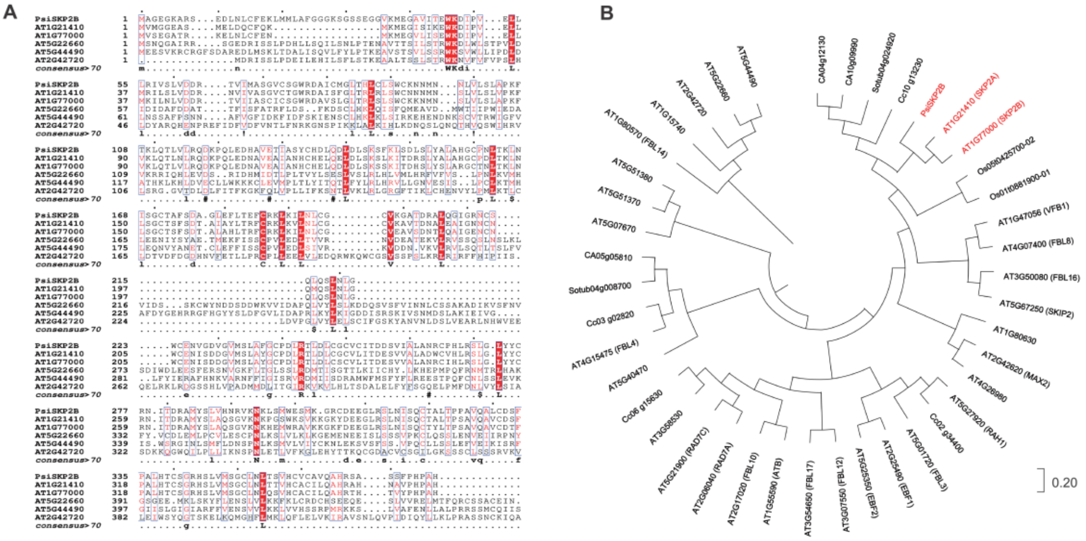

Supplementary Figure S6. Sequence identity of SKP2 proteins. A) Multiple alignment of SKP2 proteins from *Arabidopsis* and PsiSKP2B. Identical and similar amino acid residues are shaded with red. **B)** Evolutionary analysis of PsiSKP2B proteins across diverse plant species (*Arabidopsis*, *Coffea canephora*, *Capsicum annuum*, *Solanum tuberosum*, *Oryza sativa*). The branching topology of the phylogenetic tree reflects evolutionary relationships among protein sequences.

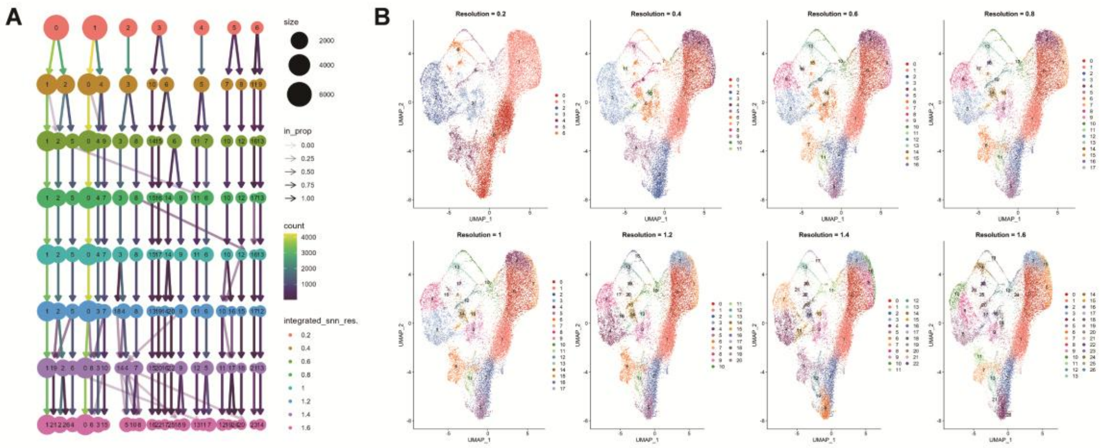

Supplementary Figure S7. The relationship between clusters at different resolutions and visualization. A) The relationship between clusters at different resolutions (0.2-1.6). The color and size of the circles indicate different resolutions and the number of cells within each cluster, respectively. Arrows, colored based on cell numbers, show the directional movement of cells between clusters. Transparency of the arrow indicates the ratio of cells in the target cluster. Greater ratios correspond to less transparent arrows. **B)** The UMAP visualization at different resolutions (0.2-1.6). Clusters are shown in different colors, and each dot represents a single cell.

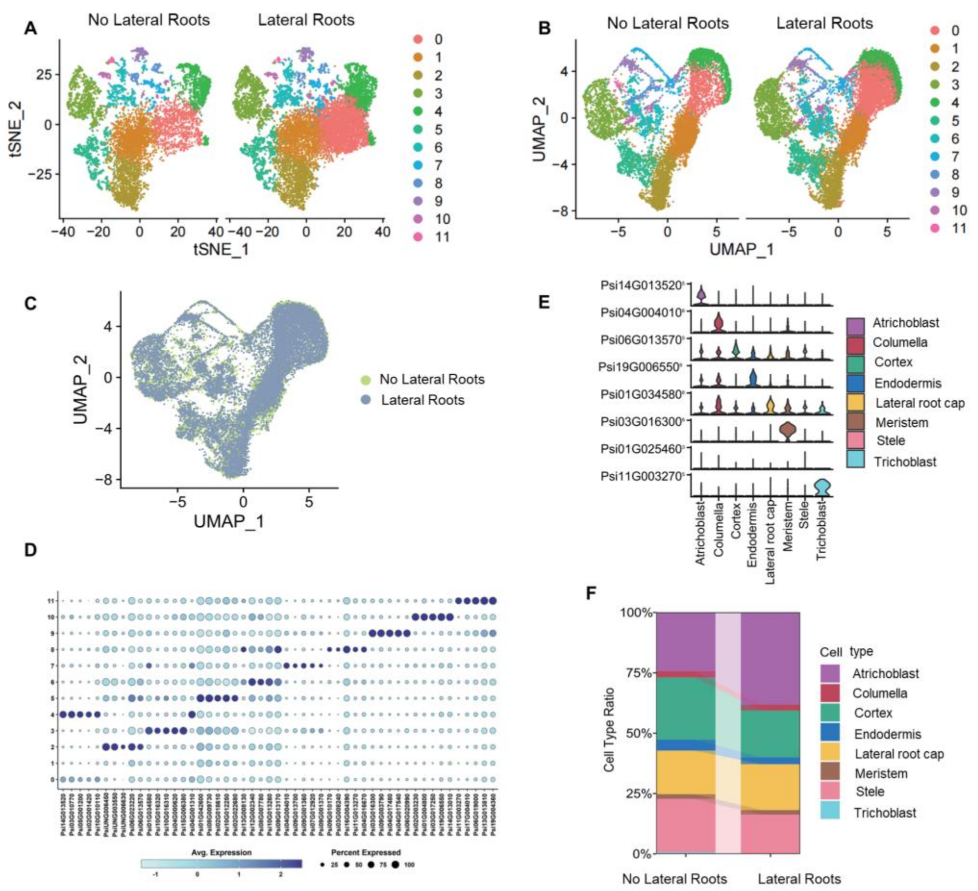

Supplementary Figure S8. Single-cell RNA-seq and cluster annotation of *Populus* root tips. A to **B)** 12 clusters are obtained by the unsupervised clustering using t-SNE **(A)** and UMAP **(B)** for lateral roots and no lateral roots sample. Clusters are shown in different colors, and each dot represents a single cell. **C)** The UMAP visualization of lateral roots and no lateral roots clusters after alignment, with each dot representing an individual cell. Green dots represent the no lateral roots sample and blue dots represent the lateral roots sample. **D)** Cluster-specific gene expression. The size of each dot represents the proportion of cells in the cluster expressing a given gene, while the color represents the mean expression level across cells in that cluster for the top five cluster-specific genes. **E)** Violin plots show the expression of marker genes in each cell type. The height of the violin represents the gene expression level, and the width of the violin represents the proportion of cells expressing in the cluster. Cell types are shown in different colors. **F)** Proportion of cell numbers per cell type in the lateral roots sample and no lateral roots sample, with each cell type color-coded.

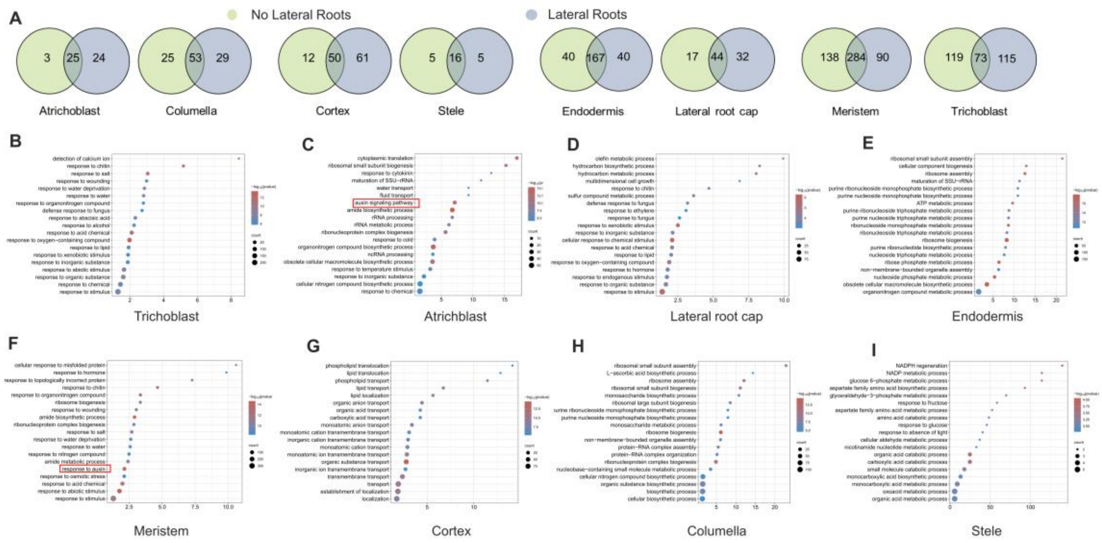

Supplementary Figure S9. Cell-type-specific genetic and functional annotations. A) Venn diagram shows the number of shared and specifically expressed genes for each cell-type cluster between the lateral roots sample and no lateral roots sample. **B** to **I)** GO enrichment analysis for each cell type. The top 20 GO term categories are shown. The y-axis shows biological processes and the x-axis indicates gene ratios. Bubble sizes represent gene numbers.

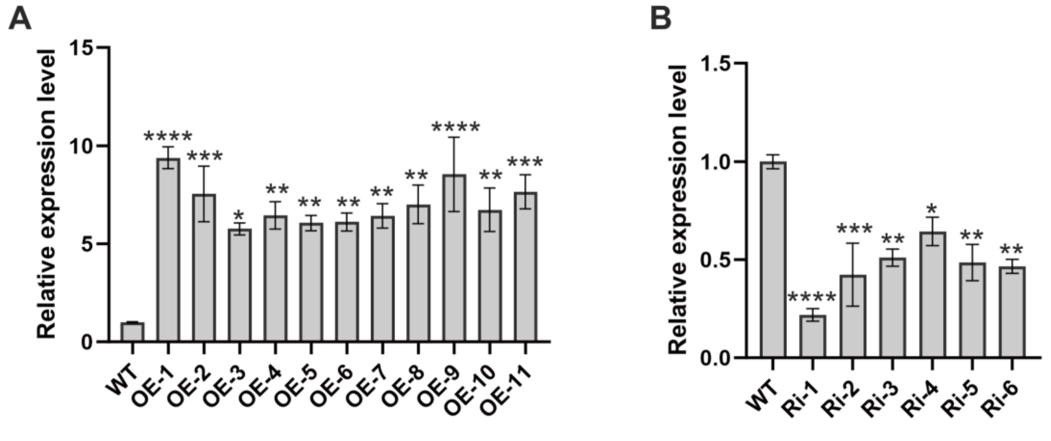

Supplementary Figure S10. Expression analysis of *PsiSKP2B* in transgenic plants. A) Expression of *PsiSKP2B* in the WT and OE plants, as determined by RT-qPCR. WT, wild-type poplar; OE, *PsiSKP2B*-overexpressing lines. Data are shown as mean ± SEM with three biological replicates. Different asterisks (*) numbers indicate statistically significant differences (one-way ANOVA followed by post hoc Tukey test; * for $P < 0.05$, ** for $P < 0.01$, *** for $P < 0.001$, and **** for $P < 0.0001$); ns, not significant. **B)** *PsiSKP2B* expression in the wild-type and *PsiSKP2B*-RNAi plants as determined by RT-qPCR. 'Ri' indicates *PsiSKP2B*-RNAi lines. Data are shown as mean ± SEM with three biological replicates. Different asterisks (*) numbers indicate statistically significant differences (one-way ANOVA followed by post hoc Tukey test; * for $P < 0.05$, ** for $P < 0.01$, *** for $P < 0.001$, and **** for $P < 0.0001$); ns, not significant.

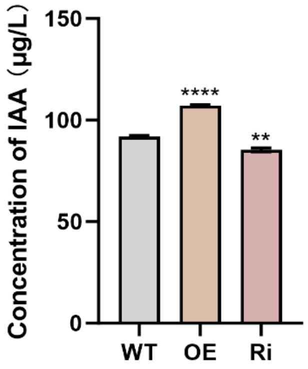

Supplementary Figure S11. IAA concentration of *PsiSKP2B* transgenic and WT plants. WT, wild-type poplar; OE, *PsiSKP2B*-overexpressing lines; Ri, *PsiSKP2B*-RNAi lines. Data are shown as mean ± SEM with three biological replicates. Different asterisks (*) numbers indicate statistically significant differences (one-way ANOVA followed by post hoc Tukey test; * for $P < 0.05$, ** for $P < 0.01$, *** for $P < 0.001$, and **** for $P < 0.0001$); ns, not significant.

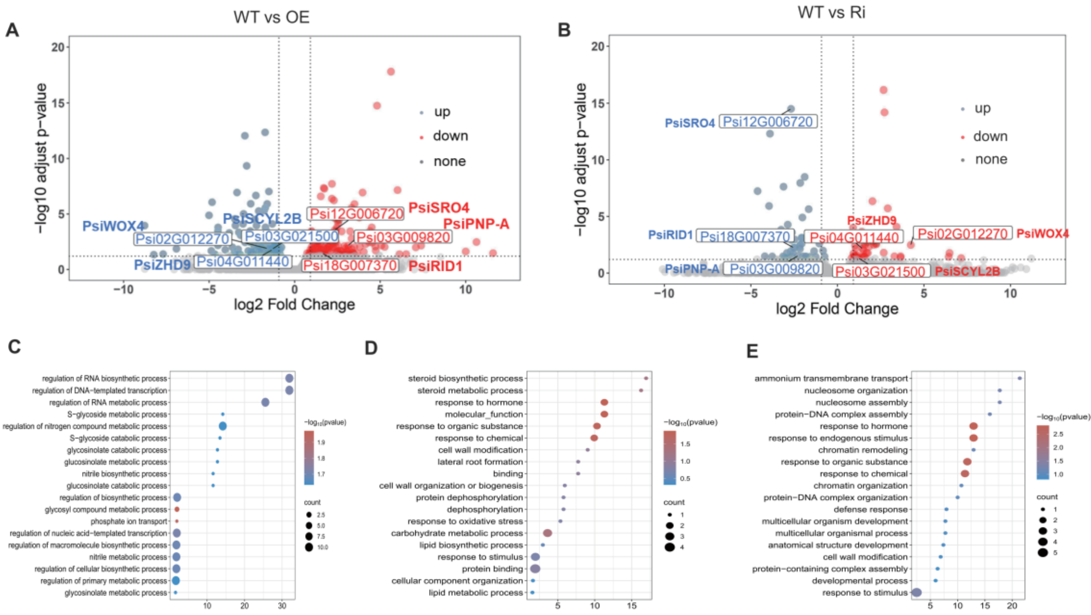

Supplementary Figure S12. GO enrichment of the DEGs in *PsiSKP2B* transgenic plant. A to B) Volcano plots show differential expression of genes between *PsiSKP2B* transgenic and WT plants ($P < 0.05$, |log2 fold change| > 1). Blue dots represent upregulated genes. Red dots represent downregulated genes, and grey dots represent genes with stable expression levels. Labels identify the three upregulated and three downregulated genes. Three DEGs are upregulated in the OE lines but downregulated in the RNAi lines, whereas another three DEGs are downregulated in the OE lines and upregulated in the RNAi lines. This pattern reveals that six DEGs are oppositely regulated in the *PsiSKP2B*-OE lines compared to the *PsiSKP2B*-Ri lines. **C)** GO enrichment of the significantly downregulated genes in *PsiSKP2B*-OE lines. **D)** GO enrichment of the significantly upregulated genes in *PsiSKP2B*-Ri lines. **E)** GO enrichment of the significantly downregulated genes in *PsiSKP2B*-Ri lines. **C to E)** The top 20 GO term categories are shown. The y-axis shows biological processes and the x-axis indicates gene ratios. Bubble sizes represent gene numbers.

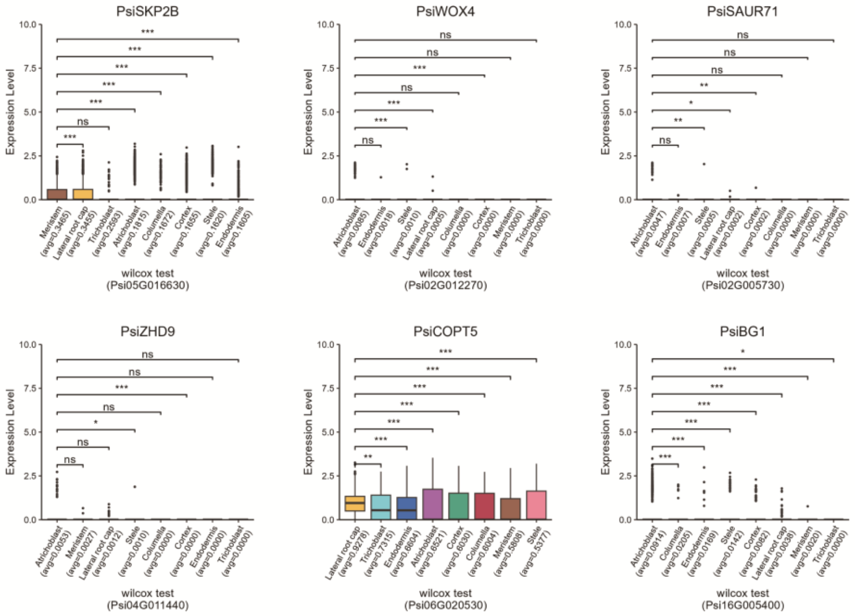

Supplementary Figure S13. The comparison of six genes (*PsiSKP2B*, *PsiWOX4*, *PsiSAUR71*, *PsiZHD9*, *PsiCOPT5*, *PsiBG1*) expression between different cell types. The data are shown as boxplots: the center line represents the median; the box limits indicate the upper (75th) and lower (25th) quartiles; the whiskers extend to 1.5 times the interquartile range from the quartiles; and points show outliers. Identified cell types are enumerated along the horizontal axis. Asterisks mark significant differences using unpaired two-sided Wilcoxon test: ns, $P > 0.05$; *, $P < 0.05$; **, $P < 0.01$; ***, $P < 0.001$. The term ‘Avg’ represents the average gene expression level in the corresponding cell type.

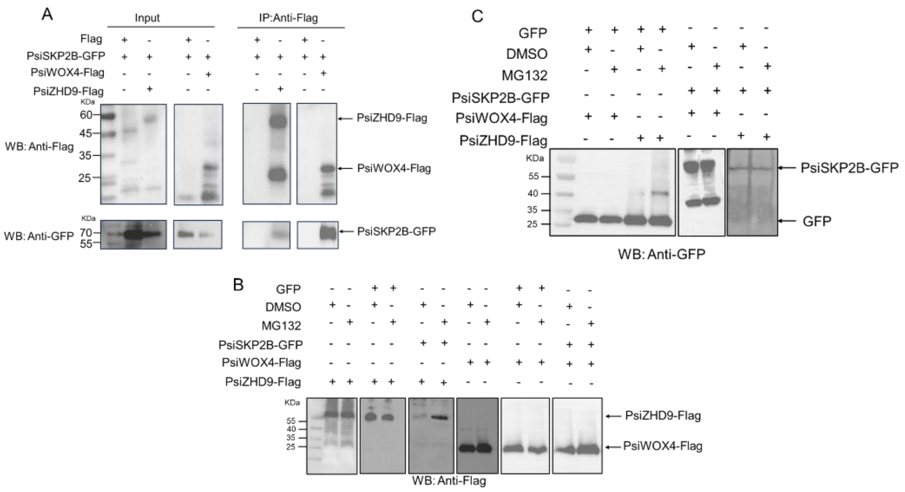

Supplementary Figure S14. PsiSKP2B ubiquitinates and targets PsiWOX4 and PsiZHD9 for degradation. A) Co-Immunoprecipitation assay for interaction of PsiSKP2B with PsiWOX4 or PsiZHD9. The total protein extracts from tobacco transfected with *PsiSKP2B*-GFP/*PsiZHD9*-Flag/*PsiWOX4*-Flag, and Flag were immunoprecipitated with anti-Flag beads. The proteins from crude lysates (left, input) and immunoprecipitated proteins (right) were detected with anti-GFP and anti-Flag antibodies. The pCAMBIA1300-FLAG and *PsiSKP2B*-GFP combination was used as a negative control. **B** to **C)** In vivo ubiquitination of PsiWOX4 and PsiZHD9 proteins by PsiSKP2B. The indicated constructs were transiently co-expressed in tobacco leaves in the presence of 50 μM MG132 or DMSO for 4 h. The *PsiWOX4*-Flag or *PsiZHD9*-Flag and GFP combination was used as a negative control. Total proteins were extracted and western blotted with anti-GFP and anti-Flag antibodies. Treatments of DMSO was used as the 'normal' buffered control.

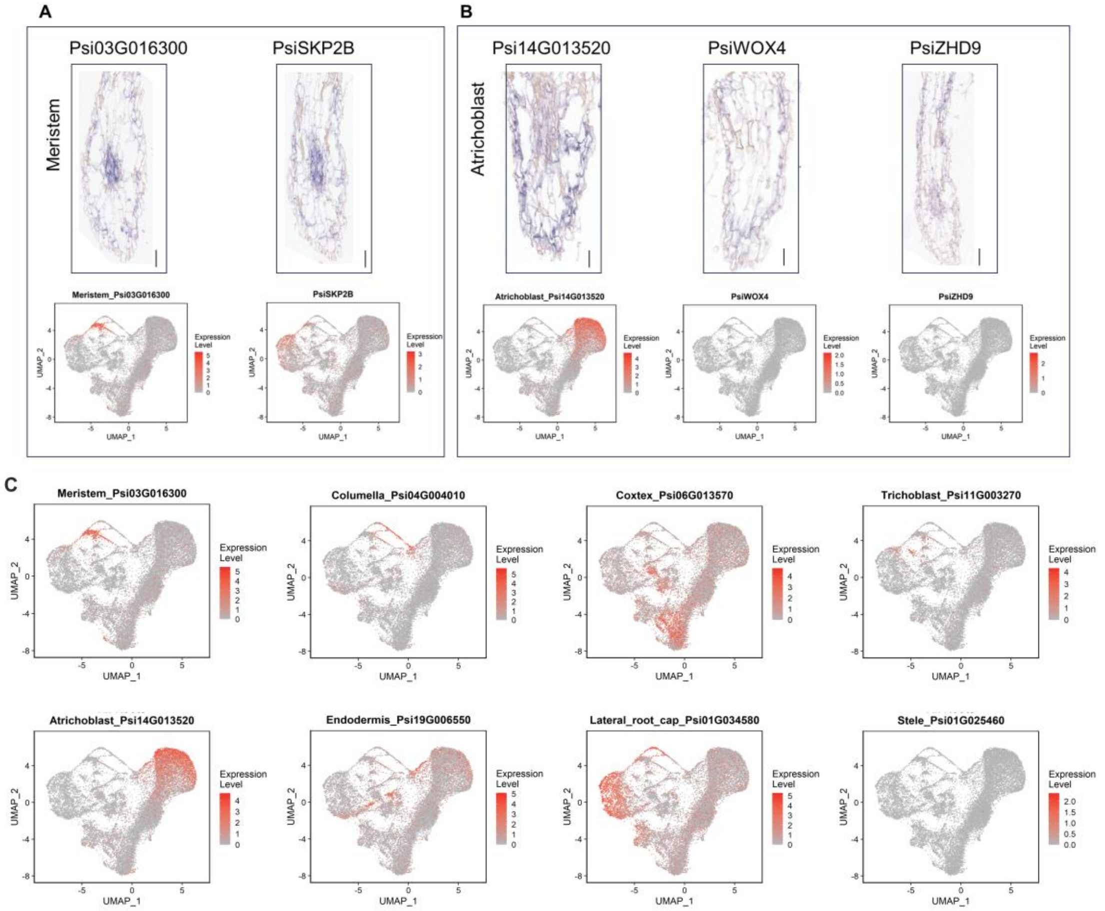

Supplementary Figure S15. RNA *in situ* hybridization detection and UMAP visualization of cell-type-specific marker genes. A to **B)** *In situ* hybridization showing that *Psi03G016300*, *PsiSKP2B*, *Psi14G013520*, *PsiWOX4* and *PsiZHD9* were specific expressed in meristematic cells and atrichoplast, respectively. Scale bars, 40 µm. The UMAP visualization presented below illustrates the distribution patterns of these five genes. Color bar indicates scaled expression level. **C)** UMAP visualization of expression of specific cell-type markers. Color bar indicates scaled expression level.
